# Embelin inhibits endothelial mitochondrial respiration and impairs neoangiogenesis during tumor growth and wound healing

**DOI:** 10.1002/emmm.201303016

**Published:** 2014-03-20

**Authors:** Oliver Coutelle, Hue-Tran Hornig-Do, Axel Witt, Maria Andree, Lars M Schiffmann, Michael Piekarek, Kerstin Brinkmann, Jens M Seeger, Maxim Liwschitz, Satomi Miwa, Michael Hallek, Martin Krönke, Aleksandra Trifunovic, Sabine A Eming, Rudolf J Wiesner, Ulrich T Hacker, Hamid Kashkar

**Affiliations:** 1 Department I for Internal Medicine, University of CologneCologne, Germany; 2 Institute for Vegetative Physiology, University of CologneCologne, Germany; 3 Institute for Medical Microbiology, Immunology and Hygiene, Medical Faculty, University of CologneCologne, Germany; 4 Department of Dermatology, University of CologneCologne, Germany; 5 Institute for Ageing and Health, Newcastle UniversityNewcastle upon Tyne, UK; 6 Cologne Excellence Cluster on Cellular Stress Responses in Aging-Associated Diseases (CECAD), Medical Faculty, University of CologneCologne, Germany; 7 Center for Molecular Medicine Cologne (CMMC)Cologne, Germany

**Keywords:** angiogenesis, embelin, mitochondria, tumor, uncoupler, wound healing, xenograft

## Abstract

In the normal quiescent vasculature, only 0.01% of endothelial cells (ECs) are proliferating. However, this proportion increases dramatically following the angiogenic switch during tumor growth or wound healing. Recent evidence suggests that this angiogenic switch is accompanied by a metabolic switch. Here, we show that proliferating ECs increasingly depend on mitochondrial oxidative phosphorylation (OxPhos) for their increased energy demand. Under growth conditions, ECs consume three times more oxygen than quiescent ECs and work close to their respiratory limit. The increased utilization of the proton motif force leads to a reduced mitochondrial membrane potential in proliferating ECs and sensitizes to mitochondrial uncoupling. The benzoquinone embelin is a weak mitochondrial uncoupler that prevents neoangiogenesis during tumor growth and wound healing by exhausting the low respiratory reserve of proliferating ECs without adversely affecting quiescent ECs. We demonstrate that this can be exploited therapeutically by attenuating tumor growth in syngenic and xenograft mouse models. This novel metabolic targeting approach might be clinically valuable in controlling pathological neoangiogenesis while sparing normal vasculature and complementing cytostatic drugs in cancer treatment.

## Introduction

In the normal quiescent vasculature of the adult, it is estimated that only 0.01% of endothelial cells (ECs) are actively proliferating. However, the proportion of proliferating ECs increases by several orders of magnitude following the angiogenic switch during tumor growth and wound healing (Tannock & Hayashi, 1972; Denekamp & Hobson, 1982). Yet, rapid tumor cell proliferation results in decreased blood perfusion because the development of supporting vasculature lags behind (Shweiki *et al*, 1992). As a consequence, the newly formed blood vessels inside the tumor experience nutrient scarcity, acidosis, and hypoxia (Hirayama *et al*, 2009). Therefore, vascular network formation in the tumor environment is dependent on the ability of ECs to survive and migrate in conditions of low nutrient availability and hypoxia. To do so, ECs need to be equipped with metabolic mechanisms to maintain sufficient energy supplies for growth and proliferation, by utilizing alternative energy substrates to compete with the metabolic requirements of tumor cells. Despite immediate access to oxygen in the blood, ECs have been reported to rely on glycolysis (Dobrina & Rossi, 1983; Krützfeldt *et al*, 1990; Mertens *et al*, 1990). However, other studies have claimed that glutamine and fatty acids were more essential substrates, suggesting an important role for oxidative phosphorylation (OxPhos) and mitochondrial respiration (Leighton *et al*, 1987; Spolarics *et al*, 1991; Dagher *et al*, 2001). How the relative contribution of OxPhos and glycolysis is regulated during angiogenesis *in vivo* is only just beginning to be understood. Indeed, recent reports indicate that the angiogenic switch is accompanied by a metabolic switch that not only regulates EC metabolism but co-determines proliferative and quiescent EC phenotypes during vessel sprouting (De Bock *et al*, 2013b). These findings illustrate an unprecedented level of metabolic control over the proliferative state in ECs during sprouting angiogenesis.

In this study, we show that the benzoquinone embelin acts on the metabolic interface between angiogenic and quiescent ECs as a weak mitochondrial uncoupler. By exhausting the low respiratory reserve of proliferating ECs, embelin impairs the angiogenic capacity of proliferating ECs during tumor angiogenesis and wound healing without adversely affecting quiescent ECs. Embelin was originally isolated from the fruit of the herbal plant *Embelia ribes* and has traditionally been used for its antitumor, antiinflammatory, and analgesic properties (Chitra *et al*, 1994). Various potential targets have been associated with its antitumor activity (Nikolovska-Coleska *et al*, 2004; Ahn *et al*, 2007; Dai *et al*, 2009; Joy *et al*, 2010); however, the antiangiogenic properties of embelin have not been explored further. Our findings point at a specific mode of mitochondrial uncoupling by benzoquinones that offers a new approach to targeting neoangiogenesis. This concept could be clinically valuable in controlling pathological neoangiogenesis while minimizing collateral damage to normal blood vessels.

## Results

### Embelin treatment inhibits tumor growth in syngenic and xenograft mouse tumor models

**Figure 1:**
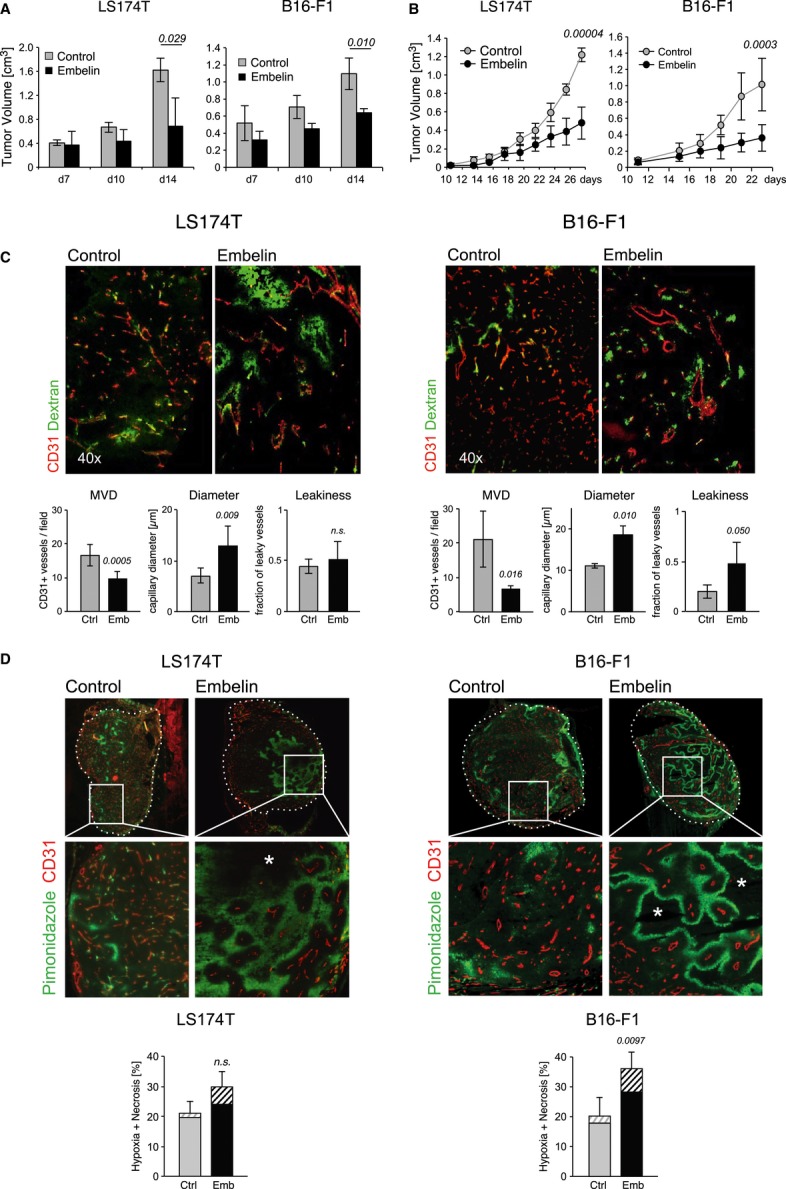
Embelin treatment attenuates tumor growth and tumor angiogenesis. A The human colorectal cancer cell line LS174T or murine melanoma cell line B16-F1 was mixed with matrigel and embelin 20 μM or vehicle (controls) and injected s.c. into recipient mice (*n* = 4). Tumor size was measured in 2 dimensions, and the calculated volume was recorded. Data points represent the mean ± s.d. B LS174T cells or B16-F1 cells were injected subcutaneously into recipient mice (*n* = 5). Embelin was injected i.p. every 48 h after palpable tumors were detectable (d10). Tumor size was measured in 2 dimensions, and the calculated volume was recorded. Data points represent the mean ± s.d. C Immunofluorescence analysis of LS174T and B16-F1 tumors after a treatment period of 2 weeks with embelin or vehicle (Control), respectively, stained for vascular endothelial cells (CD31 in red) and FITC-dextran (green). Quantification of microvessel density (MVD), vessel diameter, and leakiness. Data points represent the mean ± s.d. D Multiple image alignment of cryosections of LS174T and B16-F1 tumors treated as in (C). Areas of hypoxia were detected as pimonidazole adducts (green), and blood vessels were stained with CD31 (red). (*) marks necrotic areas. Quantification of hypoxia and necrosis (lower panel). Necrosis is represented by a striped bar. Data points represent the mean ± s.d. (*n* = 6).

To investigate the antitumor activity of embelin *in vivo*, matrigel containing embelin (20 μM) or vehicle was mixed with murine B16-F1 melanoma cells or human colon carcinoma LS174T cells and injected into the flank of recipient mice. The growth rate of these matrigel plugs was significantly attenuated in the presence of embelin (Fig 1A). In a second approach, B16-F1 or LS174T cells were subcutaneously injected into recipient mice. Animals received intraperitoneal injections of embelin (8 mg/kg) or vehicle (controls) every 48 h from the time of palpable tumor development. Embelin-treated animals showed a significant reduction in tumor growth after 3 weeks of treatment (Fig 1B).

### Embelin treatment leads to reduced microvessel density in murine tumor models

The tumor vasculature was analyzed in more detail for embelin-mediated antiangiogenic effects. Assessment of the microvessel density (MVD) in tumors of embelin-treated animals showed a striking reduction compared with vehicle-treated controls (Fig 1C). In addition, the average vessel diameter was significantly increased compared to controls, suggesting the preferential regression of smaller capillary vessels or structural vessel defects following embelin treatment (Fig 1C). Following tail vein injection of high molecular weight FITC-conjugated dextran, there was an increased leakage into the interstitial space in B16-F1 xenografted mice, while tumor vessels in LS174T tumors tended to be more leaky regardless of embelin treatment (Fig 1C). However, we found no obvious differences in pericyte coverage or basement membrane deposition between embelin-treated animals and controls (Supplementary Fig S1A). To investigate whether the reduced tumor growth was the result of increased hypoxia and cell death, the tumor vessel function was examined by injecting the hypoxia marker pimonidazole (2-nitroimidazole) into the tail vein of tumor-bearing mice. Under hypoxic conditions, pimonidazole adducts form that can be detected by immunohistochemistry. Overall, an increase in hypoxia and cell death was observed owing to the reduction in MVD in embelin-treated tumors (Fig 1D). Nevertheless, the lack of pimonidazole adducts surrounding the residual tumor blood vessels in embelin-treated animals indicated that these vessels were adequately perfused and capable of carrying oxygen (and embelin) into the nearby tumor tissue. In these perfused areas, there was also no significant reduction in tumor cell proliferation (Ki67) or increase in apoptotic cell death (caspase-3 staining), suggesting that embelin did not directly inhibit tumor cell growth (Supplementary Fig S1B). Consistent with this, matrigel plugs containing embelin (20 μM) failed to be vascularized (Supplementary Fig S1C). These analyses show that rather than targeting the tumor cells *per se,* embelin attenuated tumor growth *in vivo* by targeting tumor blood vessels leading to inadequate nutrient and oxygen supply and ultimately a greater fraction of tumor cell death/necrosis.

Recently, CD105 (endoglin) expression has been correlated with the proliferation rate of ECs in tissues participating in physiological and pathological neoangiogenesis (Fonsatti *et al*, 2003; Lebrin *et al*, 2004). We found that tumors from embelin-treated mice showed a disproportionate loss of CD105-positive ECs compared to controls, suggesting that proliferating ECs rather than established quiescent blood vessels were preferentially targeted by embelin treatment (Supplementary Fig S1D). Importantly, we found no gross morphological evidence of embelin-induced disruption of normal quiescent vasculature in experimental animals (Supplementary Fig S1E), consistent with its well-tolerated use in traditional medicine (Gupta *et al*, 1977).

To explore whether physiological processes such as wound healing that require neoangiogenesis would also be affected by embelin treatment, we investigated its effect on cutaneous wound healing.

### Embelin inhibits angiogenesis during wound healing *in vivo*

**Figure 2:**
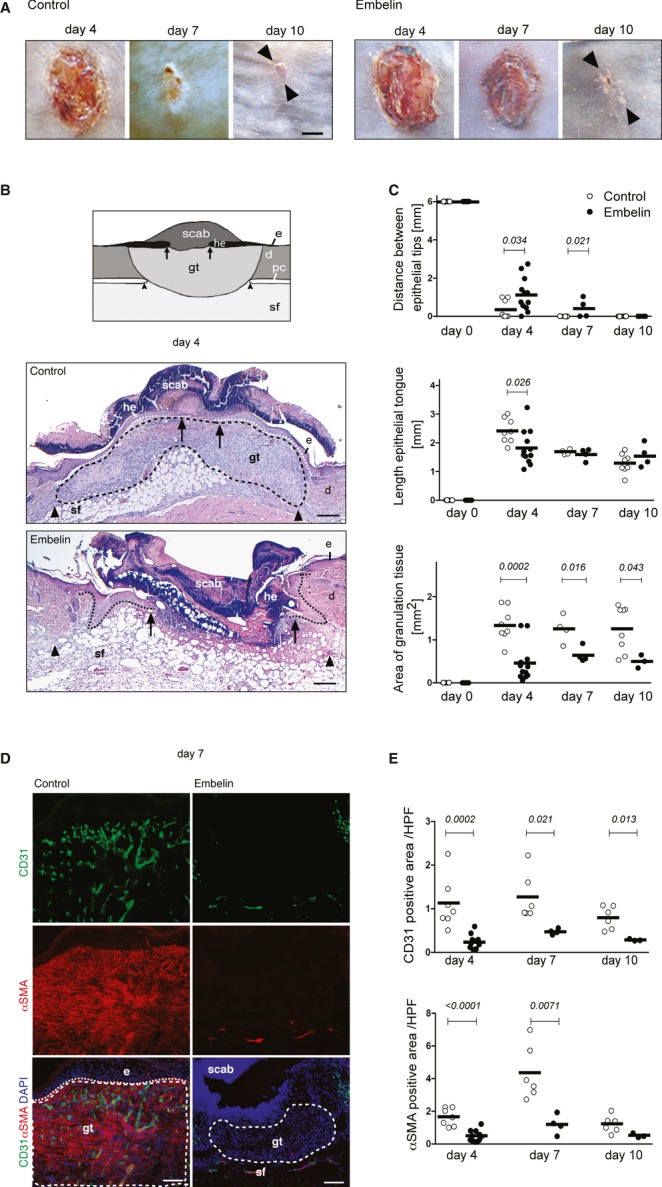
Embelin attenuates the formation of vascular granulation tissue and delays wound healing. A Punch wounds at the indicated time points post injury of embelin- or vehicle (control)-treated mice. Wounds in controls, but not embelin-treated mice, were almost completely epithelialized on day 7 post injury, whereas in embelin-treated mice wounds still carry a scab; on day 10, arrowheads indicate scar tissue; scale bar, 500 μm. B Schematic representation of wound histology on day 4 post injury and representative H&E-stained sections 4 days post injury; scale bar 100 μm. C Morphometric analysis of wound tissue at different time points post injury; each dot represents one wound. The distance between the epithelial tips, the length of the epithelial tongues, and the area of granulation tissue was measured. D Immunofluorescence staining of CD31 and α-SMA in wounds of control and embelin-treated mice on day 7 post injury. E Quantification of CD31 and α-SMA staining within granulation tissue; scale bar, 100 μm. Data information: Dashed lines outline granulation tissue; dotted line indicates epidermal–dermal junction; arrows indicate tip of epithelial tongue; arrowheads indicate ends of the injured panniculus carnosus at the wound edge; e, epidermis; he, hyperproliferative epidermis; d, dermis; gt, granulation tissue; sf, subcutaneous fat tissue.

Full-thickness circular excisional wounds were inflicted on the dorsum of C57/Bl6 mice. Animals were treated with embelin (8 mg/kg) or vehicle by intraperitoneal injection every 48 h beginning 1 day after wounding. Macroscopic wound closure was delayed in embelin-treated mice as compared to vehicle-treated animals (Fig 2A). To assess dermal repair, the amount and vascularization of granulation tissue was examined in histological sections (Fig 2B and D). Unlike the highly cellular and vascularized granulation tissue that normally develops beneath the neoepidermis, embelin-treated animals showed only scarcely vascularized granulation tissue (Fig 2D). Whereas in control mice the microvessel density within the granulation tissue increased during the healing response, in embelin-treated mice the number of vessels was dramatically reduced (Fig 2E) and endoglin-positive sprouting capillaries were almost absent (data not shown). Furthermore, the impaired development of vascularized granulation tissue in embelin-treated animals was associated with a significant reduction in α-SMA-stained myofibroblasts and pericytes (Fig 2C) and a delay in epithelialization (Fig 2B). On day 7 post injury, most control wounds appeared completely epithelialized, whereas embelin-treated wounds were still carrying a scab (Fig 2B). The distance between the tips of the epithelial tongues was significantly larger in embelin-treated animals than in controls (Fig 2B and C) and the formation of neoepidermis (i.e., the length of the epithelial tongues) was significantly reduced on day 4 after injury (Fig 2C).

These findings demonstrate that systemic treatment with embelin dramatically delayed neoangiogenesis and granulation tissue formation and attenuated wound closure kinetics. Given that the myeloid compartment can influence pathological angiogenesis, detailed histological quantifications of polymorphonuclear neutrophils (GR1^+^) and macrophages (CD68^+^) were conducted in punch wounds and in B16-F1 tumors of immunocompetent animals (Supplementary Fig S2A and B). However, no significant differences were observed in the number of infiltrating macrophages or PMNs in response to embelin treatment, making it unlikely that the antiangiogenic effects of embelin treatment in wounds and tumors are secondary to alterations in PMN and macrophage infiltration. Collectively, these observations in the wound healing model corroborate the potent antiangiogenic effects of embelin observed during tumor angiogenesis.

### Embelin treatment targets ECs under growth condition

**Figure 3:**
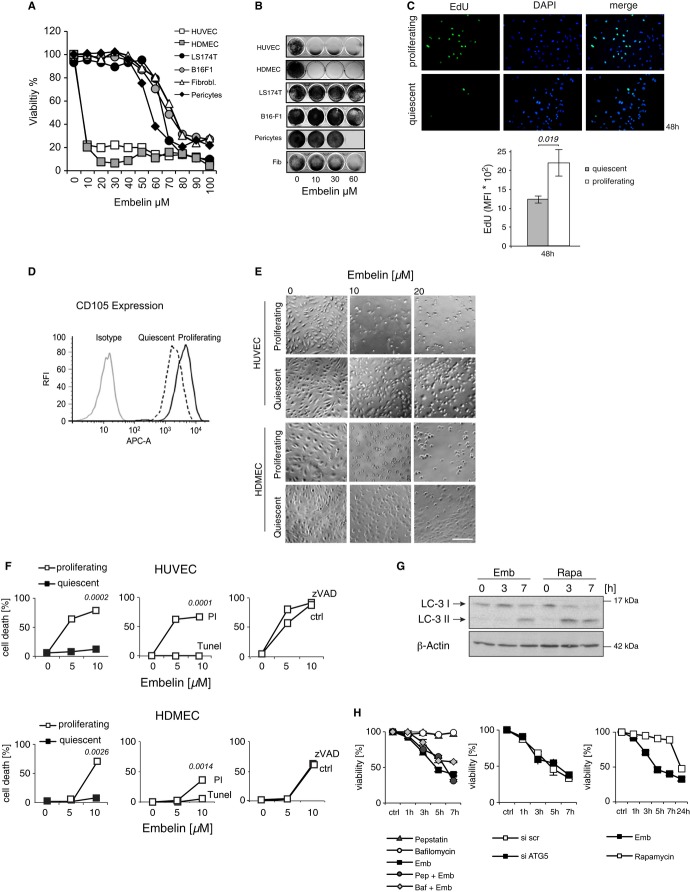
Embelin treatment disrupts endothelial cell function and survival *in vitro.* A Measurement of cell viability of human umbilical vein endothelial cells (HUVECs), human dermal microvascular endothelial cells (HDMECs), LS174T and B16-F1, fibroblasts and pericytes in response to embelin treatment. Viability was assessed by trypan blue exclusion after embelin treatment for 3 h. B Measurement of clonogenicity of cell lines as in (A). Clonogenicity was assessed by crystal violet staining of adherent colonies 14 days after exposure to embelin. C Detection of EdU incorporation in proliferating and quiescent HUVECs. Proliferating cells are stained green. Nuclei were counterstained with DAPI (blue, upper panel). Quantification of EdU incorporation by FACS analysis. Data points represent the mean ± s.e.m. (*n* = 3; lower panel). MFI, mean fluorescence intensity; scale bar, 200 μm. D FACS analysis of CD105 expression in quiescent and proliferating HUVECs. RFI, relative fluorescence intensity. E Phase-contrast microscopy images of quiescent and proliferating cultures of HUVECs and HDMECs after embelin treatment for 3 h; scale bar, 200 μm. F Cell death was measured by trypan blue exclusion assay in proliferating and quiescent HUVECs or HDMECs (left panel). Propidium iodide (PI) and TUNEL staining were measured in proliferating HUVECs and HDMECs in response to embelin by FACS analysis (middle panel). Cell death was measured by trypan blue exclusion in the presence or absence of zVAD (right panel; *n* = 3). G LC-3 conversion was analyzed in total cell lysates after treatment with embelin (5 μM) or rapamycin (40 mg/ml) at the indicated time points. H Cell death measured by trypan blue exclusion in proliferating HUVECs following treatment with embelin (5 μM), pepstatin (10 μg/ml), bafilomycin (160 nM), or combinations as indicated (left panel), or following specific knockdown of ATG5 or transfection with scrambled (scr) siRNA (center panel), or following treatment with embelin (5 μM) or rapamycin (40 mg/ml; right panel; *n* = 3). Source data are available online for this figure.

To better define the target cell population of embelin, we compared its effect on ECs and non-ECs *in vitro*. The viability and clonogenicity of primary EC cultures of human umbilical vein endothelial cells (HUVECs) and human dermal microvascular endothelial cells (HDMECs) was reduced at embelin concentrations far below those required to induce similar effects in non-ECs such as tumor cells and stromal cells including pericytes and fibroblasts (Fig 3A and B, Supplementary Fig S3A and B).

Unlike proliferating blood vessels found in tumor tissue or during wound healing, most established normal blood vessels are comprised of ECs that are growth quiescent (Hanahan & Folkman, 1996). Our data summarized in Fig 1 and Supplementary Fig S1 indicate a specific cytotoxic effect of embelin toward angiogenic, but not quiescent, ECs. To investigate this cytotoxic property of embelin on proliferating versus quiescent ECs, quiescence was induced in contact-inhibited confluent EC cultures of HUVECs and HDMECs by growth factor depletion for 24 h as described previously (Mahboubi *et al*, 2001; Kurz *et al*, 2003; Adams *et al*, 2005; Mariotti *et al*, 2006; Vag *et al*, 2009). The quiescent phenotype was confirmed by appropriate changes in morphology, up-regulated VE-cadherin expression, cell cycle arrest, reduced EdU incorporation, and down-regulation of KI67 and CD105 expression (Fig 3C and D, Supplementary Fig S3C and F). When exposed to embelin, only ECs under growth conditions underwent rapid detachment and cell death, whereas the quiescent ECs were refractory even after exposure for 24 h (Fig 3E, Supplementary Fig S3G). The observed cytotoxicity was non-apoptotic, as demonstrated by the lack of TUNEL staining, caspase cleavage, and cytochrome c release (Fig 3F and Supplementary Fig S3H and I). Accordingly, cell death could not be inhibited by the pan-caspase inhibitor zVAD or the inhibitor of caspase-independent programmed necrosis necrostatin (Fig 3F and Supplementary Fig S3J). Finally, we investigated whether embelin induced autophagic cell death. Indeed, we found that embelin treatment of ECs leads to the conversion of LC3-I to LC3-II (Fig 3G and Supplementary Fig S3K); however, embelin-induced cell death was only minimally affected when autophagy was inhibited with bafilomycin or pepstatin, or by specific knockdown of ATG5 (Fig 3H). Furthermore, embelin-induced cell death preceded rapamycin-induced autophagic cell death by almost 20 h (Fig 3H), suggesting that the induction of autophagic cell death is not a major cytotoxic effect of embelin. In summary, these data indicated that embelin treatment preferentially induced non-programmed cell death in proliferating but not in quiescent ECs, stromal, or tumor cells.

### Embelin is a weak mitochondrial uncoupler that selectively targets proliferating ECs

Although a range of cellular targets have previously been reported for embelin's antitumor activity—including ROS production (Allensworth *et al*, 2012), suppression of NF-κB (Ahn *et al*, 2007), and inhibition of XIAP (Nikolovska-Coleska *et al*, 2004)—at the comparatively low concentrations required to induce EC cell death, embelin did not induce ROS production (Supplementary Fig S4A), did not alter NF-κB activity (Supplementary Fig S4B) nor did it affect XIAP levels (Supplementary Fig S4C). In fact, siRNA-mediated XIAP knockdown in ECs did not induce EC death.

**Figure 4:**
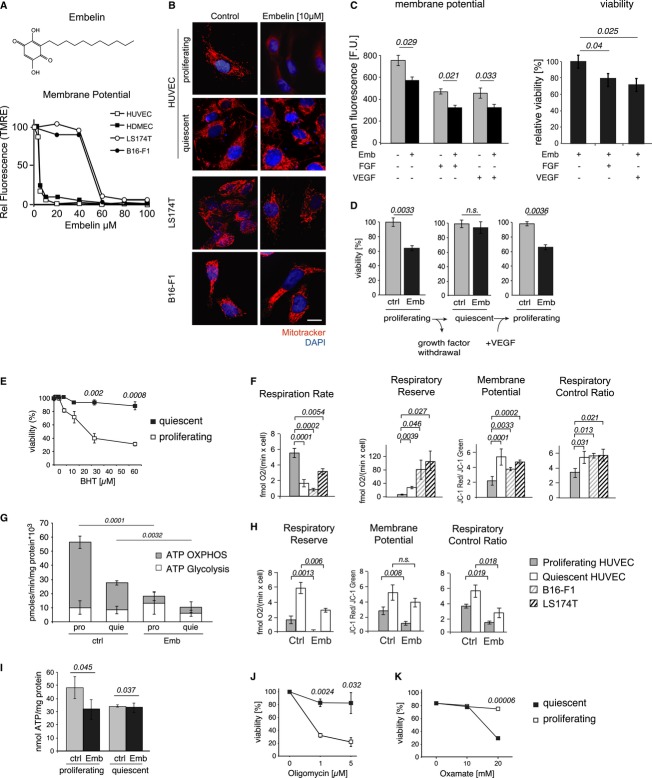
Embelin is a mitochondrial uncoupler that specifically targets proliferating endothelial cell. A Structure of the benzoquinone embelin and quantification of the mitochondrial membrane potential (MMP) in proliferating human umbilical vein endothelial cells (HUVECs), human dermal microvascular endothelial cells (HDMECs), LS174T, and B16-F1 cells in response to embelin by FACS analysis of TMRE-stained cells. B Fluorescence analysis of proliferating or quiescent HUVECs, LS174T, and B16-F1 in response to embelin, stained with MitoTracker-Red. Nuclei were counterstained with DAPI; scale bar, 20 μm. C Quantification of the MMP (left panel) and cell death (right panel) in proliferating HUVECs stimulated with VEGF (20 ng/ml) or FGF (20 ng/ml) or embelin (5 μM) for 24 h and analyzed by FACS of TMRE-stained cells or trypan blue exclusion, respectively (*n* = 3). D Quiescent HUVECs were stimulated overnight with VEGF (20 ng/ml). Cell death was measured in proliferating, quiescent, and “restimulated” proliferating cells in response to embelin (5 μM). Data points represent the mean ± s.e.m. (*n* = 3). E Measurement of cell viability of proliferating and quiescent HUVECs in response to the indicated concentrations of butylated hydroxytoluene (BHT). Viability was assessed by trypan blue exclusion. Data points represent the mean ± s.e.m. (*n* = 3). F Respiratory activity in proliferating and quiescent HUVECs, LS174T, and B16-F1 (left panel) and indices of mitochondrial performance (respiratory reserve, MMP, respiratory control ratio; *n* = 3). G ATP production by oxidative phosphorylation (OxPhos) or glycolysis was measured in proliferating and quiescent HUVECs with or without embelin (5 μM) treatment for 30 min (*n* = 3). H Impact of embelin treatment on mitochondrial performance of proliferating versus quiescent HUVECs (*n* = 3). I ATP content was measured in proliferating and quiescent HUVECs with or without embelin (5 μM) treatment for 50 min (30 min at 37°C, 20 min at RT; *n* = 3). J Measurement of cell viability of proliferating and quiescent HUVECs in response to the indicated concentrations of oligomycin. Viability was assessed by trypan blue exclusion (*n* = 3). K Measurement of cell viability of proliferating and quiescent HUVECs in response to the indicated concentrations of oxamate. Viability was assessed by trypan blue exclusion (*n* = 3).

However, previous biochemical studies have shown that benzoquinones including embelin can act as uncouplers of the mitochondrial membrane potential (MMP; Makawiti *et al*, 1990). We therefore measured changes in the MMP in response to increasing embelin concentrations and found that proliferating ECs were readily depolarized by low concentrations of embelin, whereas quiescent ECs and tumor cells were not readily uncoupled (Fig 4A and B and Supplementary Fig S4D). Interestingly, EC stimulation with pro-angiogenic growth factors (VEGF or FGF) further lowered the intrinsic MMP, thus sensitizing them to embelin-mediated uncoupling and cell death (Fig 4C). Furthermore, embelin-induced cell death could be restored even in quiescent ECs by re-exposing them to growth factors underscoring the specific cytotoxicity of embelin toward proliferating ECs (Fig 4D).

Of note, detailed time-course analysis demonstrated that the depletion of the MMP clearly preceded cell death and was therefore not a consequence of it (Supplementary Fig S4E). Furthermore, another weak mitochondrial uncoupler, butylated hydroxytoluene (BHT), also potently induced cell death in ECs under growth conditions only (Fig 4E). Together, these data suggest that differences in the mitochondrial respiratory performance of proliferating versus quiescent ECs could be underlying their differential sensitivity toward embelin.

We therefore examined the mitochondrial respiratory chain performance. Both the basal and the specific respiration rates through complexes I, II, and II/III were higher in proliferating than in quiescent ECs and tumor cells (Fig 4F and Supplementary Fig S4F and G), indicating that proliferating ECs have a higher energy demand, which they cover in large part by OxPhos. Under proliferating conditions, HUVECs and HDMECs consumed three times more oxygen than under basal conditions. Conversely, the respiratory reserve, that is, the ability of electron transport to respond to a further increase in energy demand, was lowest in proliferating HUVECs, suggesting that they already operated near their bioenergetic limit (Fig 4F). The lower inner membrane potential and low respiratory control ratio measured in proliferating HUVECs is a direct consequence of the greater utilization of the proton motif force by the ATP synthase, as similar proton leak values were observed for all cell types, indicating similar coupling (Supplementary Fig S4H). Indeed, twice as much ATP was produced in proliferating ECs compared to quiescent ECs, and a much greater proportion (> 80%) of the ATP pool was produced by OxPhos in proliferating ECs (Fig 4G). In contrast, glycolytic ATP production in both cell types was not significantly different.

Embelin treatment resulted in a further drop in the respiratory control ratio and the MMP in both proliferating and quiescent HUVECs; however, the complete depletion of the respiratory reserve was only observed in proliferating ECs (Fig 4H). Following embelin treatment, OxPhos-dependent ATP production became negligible and the ATP content in proliferating ECs dropped to the level found in quiescent ECs (Fig 4I). Despite the potential for increased glycolytic ATP production (Supplementary Fig S4I), there was no significant compensatory increase in glycolytic ATP production as illustrated by a negligible increase in extracellular acidification (ECAR) after embelin treatment (Fig S4J). Thus, the increased utilization of the proton motif force combined with the limited respiratory reserve predisposes proliferating ECs to metabolic exhaustion and cell death upon mitochondrial uncoupling. Indeed, similar effect could be observed when mitochondrial ATP synthase was blocked with oligomycin (Fig 4J). These findings suggest that the embelin-induced cell death of ECs is the result of the inability of the mitochondria to meet ATP demand under growth conditions.

The increase in respiratory activity observed in proliferating ECs compared with quiescent ECs prompted us to investigate the relative dependence on glycolysis using oxamate, a substrate-like inhibitor of lactate dehydrogenase (LDH), the enzyme that converts pyruvate to lactate (Papaconstantinou & Colowick, 1961). Oxamate treatment abolished lactate production in HUVECs (Supplementary Fig S4K) and induced significant cell death in quiescent but not in proliferating ECs, demonstrating that quiescent ECs are more dependent on glycolytic metabolism while illustrating the capacity of proliferating ECs for mitochondrial metabolism of pyruvate via the Krebs cycle (Fig 4K).

### Mitochondrial dysfunction is associated with disrupted angiogenesis in the mitochondrial DNA mutator mouse model

**Figure 5:**
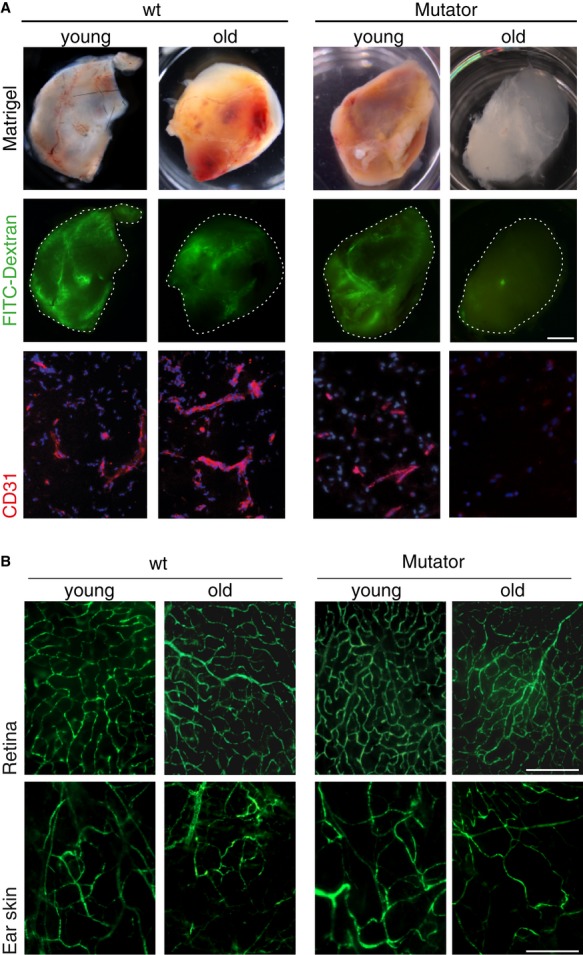
Neoangiogenesis is impaired in mtDNA mutator mice. A Matrigel was injected s.c. into recipient control and mutator mice 12 weeks (young) or 30 weeks (old) of age. Photograph of the plug after 11 days (upper panel). FITC-dextran is shown in green (middle panel). Microscopic analysis of CD31 (red) and DAPI (lower panel); scale bar, 5 mm. B Microscopic analysis of FITC-dextran in retina and ear skin of young (12 weeks) or old (30 weeks) wild-type and mutator mice treated as in (A); scale bar, 200 μm.

To independently demonstrate the important role of functional mitochondrial OxPhos in neoangiogenesis, we employed a genetic mouse model for mitochondrial dysfunction and aging (Trifunovic *et al*, 2004). This mouse model was engineered to have a defect in the proofreading function of the catalytic subunit of mitochondrial DNA (mtDNA) polymerase (POLGA), leading to the progressive, random accumulation of mtDNA mutations associated with impaired assembly and stability of the respiratory chain complexes and a premature aging phenotype (Edgar *et al*, 2009). The first premature aging symptoms, for example, slight kyphosis, alopecia, and impaired weight gain, can be observed in mtDNA mutator mice beginning at 25 weeks of age (Trifunovic *et al*, 2004). By contrast, at 12 weeks of age, detailed and systematic assessment of mitochondrial respiratory activity revealed no obvious alteration (Trifunovic *et al*, 2004). To investigate neoangiogenesis in these mice, matrigel supplemented with VEGF and FGF was injected subcutaneously into young (12-week-old) and aged (30-week-old) mtDNA mutator mice and age-matched young and old wild-type mice. The animals were injected with FITC-dextran after 11 days and matrigel plugs were harvested. Macroscopic analysis showed that matrigel plugs from young mutator mice aged between 10 and 12 weeks were as efficiently vascularized as matrigel plugs from wild-type animals (including young and old animals; Fig 5A upper panel). Furthermore, the endothelial networks formed in these plugs have a functional lumen capable of conducting FITC-labeled dextran into the matrigel plug (Fig 5A middle panel). In contrast, matrigel plugs implanted into aged mutator mice (30 weeks) were barely vascularized after 11 days, as evident from the macroscopic appearance and lack of FITC-dextran signal. Histological analysis for CD31 expression confirmed the absence of new capillaries in the aged mtDNA mutator mice, in contrast to the presence of new capillaries in plugs from young mutator and wild-type animals (Fig 5A lower panel). To complement these analyses, we also show that established vascular networks in retina and ear skin were not disrupted in old mutator mice consistent with the specific dependency of neovessel formation on mitochondrial function (Fig 5B). Together, these findings independently support the concept that neoangiogenesis is critically dependent on mitochondrial function and that disruption of mitochondrial function by pharmacological or genetic means can be used to therapeutically target neoangiogenesis.

## Discussion

Tumor growth and wound healing require the temporary activation of quiescent ECs for the formation of new blood vessels. Unlike quiescent ECs, angiogenic ECs are engaged in a number of energy-consuming biological processes, such as proliferation, migration, and capillary tube formation that may require metabolic adaptation to cover the increasing energy demand. Indeed, recent evidence suggests that a metabolic switch accompanies the proliferative response during angiogenesis (De Bock *et al*, 2013a). In this study, we demonstrated that neoangiogenesis can be inhibited in mouse models of tumor growth and cutaneous wound healing by targeting mitochondrial oxidative metabolism both pharmacologically and in a genetic mouse model.

All cells must respond to changes in the availability of substrates for energy metabolism in order to survive. When glucose is scarce—as can occur in solid tumors—ECs are forced to change from generating ATP predominantly by glycolysis to OxPhos using alternative energy substrates that can be oxidized by the mitochondria including glutamine, lactate (Parra-Bonilla *et al*, 2010), and fatty acids (Dagher *et al*, 2001). Oxidative metabolism in ECs is inhibited at physiological concentrations of glucose, a phenomenon known as the Crabtree effect (Krützfeldt *et al*, 1990), however, this is not the case at the low glucose levels experienced in the tumor microenvironment (Hirayama *et al*, 2009). Indeed, we show that proliferating HUVECs and HDMECs consume three times more oxygen than quiescent ECs and work close to their respiratory limit. It has previously been demonstrated that in addition to serving as a conduit for the delivery of oxygen, the microvasculature itself consumes a significant amount of oxygen (Tsai *et al*, 1998). To sustain EC proliferation in the absence of extracellular glucose, lactate secreted from glycolytic tumor cells is sufficient to restore ATP levels and partially rescue EC growth, suggesting that lactate is converted into pyruvate for mitochondrial respiration (Parra-Bonilla *et al*, 2010). In addition, fatty acid oxidation can support EC proliferation and the disruption of fatty acid handling limits EC proliferation (Elmasri *et al*, 2009). But even in the absence of exogenous substrates, ECs can oxidize endogenous amino acids or lipids (Mertens *et al*, 1990; Dagher *et al*, 2001). Consistent with an increased utilization of OxPhos, proliferating ECs become less dependent on glycolytic elimination of pyruvate. This is illustrated by our finding that oxamate, a substrate inhibitor of LDH, induced significant cell death in quiescent, but not in proliferating, ECs (Fig 4K). LDH permits the rapid consumption of pyruvate, regenerating NAD^+^ to sustain the glycolytic flux while yielding a product (lactate) that can easily be secreted. This permits the generation of glycolytic energy when OxPhos activity is low by avoiding pyruvate accumulation. Therefore, the susceptibility to LDH inhibitors is increased in cells with predominantly glycolytic metabolism, whereas cells with increased OxPhos activity are independent of LDH activity as they can feed pyruvate into the Krebs cycle.

Our detailed metabolic analysis of embelin identifies it as a *bona fide*, however, weak uncoupler of the MMP, which is capable of dissociating electron flow from ATP synthesis, thus exhausting the already limited respiratory reserve of proliferating ECs. Importantly, other weak mitochondrial uncouplers such as BHT also induce cell death in proliferating but not in quiescent ECs, further supporting the evidence that the antiangiogenic activity of embelin is due to its activity as a mitochondrial uncoupler. While the use of mitochondrial uncouplers in patients is not unprecedented, the narrow therapeutic window of conventional uncouplers led to the abandonment of their official use in the treatment of obesity (Harper *et al*, 2001). However, BHT has been shown to partially uncouple mitochondria over a wide dynamic range (the difference between therapeutic and toxic doses), thus presenting a more promising therapeutic opportunity (Lou *et al*, 2007).

The transition from resting (slow respiring) state 4 to phosphorylating (fast respiring) state 3 is associated with an increased utilization of the proton motif force by the mitochondrial ATP synthase leading to a decrease in the MMP (Chance & Williams, 1956; Yuan *et al*, 1996). Accordingly, pro-angiogenic growth factors such as VEGF or FGF induce a further reduction in the intrinsic MMP in proliferating ECs that predisposes them to mitochondrial uncoupling and cell death (Fig 4C). Remarkably, quiescent ECs can be re-sensitized to embelin-induced cell death by exposure to angiogenic growth factors, as might occur in the context of tumor growth or wound healing (Fig 4D). Consistent with this, embelin preferentially targets proliferating endoglin-expressing blood vessels rather than mature quiescent vasculature (Supplementary Fig S1D). In contrast, most normal blood vessels under physiological conditions are growth quiescent and therefore resistant to embelin treatment. From a clinical perspective, this is important because the selective targeting of neoangiogenesis could help to restrict tumor growth without adversely impacting on quiescent normal blood vessels or mature tumor vessels potentially required for the delivery of cytostatic chemotherapy.

Sprouting blood vessels are made up of different cell populations: leading tip cells, which guide the sprout, and trailing stalk cells, which proliferate to elongate the stalk (De Smet *et al*, 2009). Recently, De Bock *et al* showed the important role of glycolytic metabolism in sprouting angiogenesis. Specifically, overexpression of the glycolytic activator PFKFB3 could induce sprouting tip cell behavior even in proliferating stalk cells (De Bock *et al*, 2013b). This is a remarkable finding, because it shows that metabolic regulators are directly involved in EC phenotype decisions, demonstrating an unprecedented degree of metabolic control during angiogenesis. In contrast to tip cells, PFKFB3 expression, and therefore glycolytic energy production, is normally inhibited in proliferating stalk cells by Notch activation (De Bock *et al*, 2013b), suggesting that alternative energy sources such as OxPhos may be employed to cover the increasing energy demand during EC proliferation. Accordingly, oxamate failed to induce cell death in proliferating ECs (Fig 4K), whereas the inhibition of mitochondrial OxPhos with oligomycin or uncoupling of mitochondria with embelin or BHT leads to the depletion of ATP (Fig 4I) and cell death in proliferating, but not in non-proliferating, ECs (Fig 3F, 4E and J). Interestingly, the proliferating endothelial stalk cells express high levels of the metabolic sensor SIRT1 (Potente *et al*, 2007) and SIRT1 is also expressed at elevated levels in proliferating rather than in quiescent HUVECs along with other regulators of OxPhos (Supplementary Fig S4L). SIRT1 activation redirects cellular metabolism from glycolysis to OxPhos by deacetylating and activating transcription factors and cofactors, such as peroxisome proliferator-activated receptor-γ coactivator-1α (PGC-1α) (Rodgers *et al*, 2005). Therefore, tip and stalk cells may use different energy production pathways. Balancing between glycolytic and mitochondrial energy production, regulated by Notch and SIRT1, might be critical in the proliferating stalk cells, whereas glycolytic energy production appears to be predominant in the migrating tip cells (Harjes *et al*, 2012).

Further evidence for the critical role of functional mitochondrial OxPhos during neoangiogenesis is provided by the impairment of neovascularization in matrigel plugs in mtDNA mutator mice. These mice serve as models of mitochondrial dysfunction and aging as they express defective mtDNA polymerase and progressively accumulate mutations in mtDNA. Measurable alterations in the mitochondrial respiratory activity start occurring after 25 weeks of age (Trifunovic *et al*, 2004). Prior to 25 weeks of age, there is no apparent phenotype or tissue defect in these mice (Trifunovic *et al*, 2004, 2005; Niu *et al*, 2007). Given that all of the proteins encoded by mitochondrial genes are involved in OxPhos (Anderson *et al*, 1981), it is tempting to speculate that the failure to recruit vascular ECs to the matrigel plugs is due to energetic collapse and that functional mitochondrial activity is necessary for the formation and/or recruitment of new blood vessels (Fig 5). While the “mutator” model is not without limitations and systemic tissue dysfunction (even if not obvious at this age) might in principle contribute to the phenotype observed in these animals, bone marrow-derived myeloid cells that have an important role in regulating the formation and maintenance of blood vessels in tumors appear to be unaffected in this mouse model (Norddahl *et al*, 2011). Furthermore, despite severe respiratory chain dysfunction, the premature aging phenotype is not caused by alterations in ROS production or oxidative stress, both potential drivers of angiogenesis (Trifunovic *et al*, 2005).

While recently the important role for glycolysis has been explored in sprouting angiogenesis, our findings demonstrate that other metabolic pathways also influence vessel formation. Specifically, mitochondrial targeting of proliferating ECs could be clinically valuable to control pathological neoangiogenesis and complement cytostatic drugs in cancer treatment, while minimizing collateral damage to normal quiescent ECs.

## Materials and Methods

### Cell lines, cell culture

The human colon carcinoma cell line LS174T, the murine melanoma cell line B16-F1, and the human colonic epithelial cell line (CoTr) were purchased from ATCC (Bethesda, Maryland, USA); human primary melanoma cells (MOO1 and HOM1) and human dermal fibroblasts were a gift of Dr C. Mauch (Department of Dermatology, University of Cologne, Germany). LS174T, MOO1, and HOM1 cells were maintained in RPMI 1640 supplemented with 10% fetal calf serum (FCS), 2 mM l-glutamine, 100 μg/ml streptomycin, and 100 U/ml penicillin (all from Biochrom, Berlin, Germany). B16-F1, CoTr, and fibroblasts were maintained in DMEM supplemented with 10% FCS, 2 mM l-glutamine, 100 μg/ml streptomycin, and 100 U/ml penicillin (Biochrom). HUVECs, HDMECs, and human pericytes (hPC-PL) were purchased from Promocell (Heidelberg, Germany) and maintained in EC growth medium between passages 2–5. Quiescence was induced in confluent HUVECs and HDMECs by cultivation in basal EC medium depleted of growth factors (Promocell) for 36 h.

### Ethics statement

All animal experiments were performed in accordance with the German animal protection law. Institutional Animal Care and Use Committee approval was obtained for all animal studies.

### Animal tumor models

C57BL6/J and Balb/cA nude mice were purchased from Charles River (Sulzfeld, Germany). Human colorectal cancer cells LS174T (3 × 10^6^) or murine melanoma cells B16-F1 (1 × 10^6^) were injected subcutaneously into the flank region of 6- to 8-week-old BALB/cA nude mice or C57/Bl6, respectively. In the treatment group, embelin 8 mg/kg (Biotrend, Cologne, Germany) was freshly dissolved in vehicle (10% DMSO, 100 mM Tris–HCl, pH 7.4) and administered by intraperitoneal injection every 48 h. The control group received injections of vehicle only. For the matrigel plug assay, 400 μl of chilled matrigel (BD Bioscience, Heidelberg, Germany) was mixed with 3 × 10^6^ LS174T or 1 × 10^6^ B16-F1 tumor cells and embelin (at a final concentration of 20 μM) at 4°C for subcutaneous injection into recipient mice. Tumor size was measured every other day using precision calipers. Tumor volume was calculated as length × width^2^ × π/6 and expressed as average ± s.e.m. Animals were housed in the animal care facility of the University of Cologne under standard pathogen-free conditions with a 12-h light/dark schedule and provided with food and water *ad libitum*.

### Mutator mouse experiments

Four hundred microliters of chilled matrigel (BD Bioscience) was injected into 12- or 30-week-old mutator mice (Trifunovic *et al*, 2004). After 11 days, mice were sacrificed and the matrigel was removed, fixed in 4% paraformaldehyde (PFA) for microscopic analysis or prepared for further downstream analyses.

### Images of matrigel plugs

Images were taken using a motorized Leica M165 FC fluorescent stereomicroscope equipped with DFC490 CCD camera and GFP2 (Ex. 480/40 nm) filter set. Images were processed using the Multifocus module of LAS 3.7.0 software (Leica).

### Cutaneous wound healing assay

Four full-thickness paravertebral punch-biopsy wounds were induced on the shaved dorsum of 12-week-old C57BL6/J mice under Ketanest/Rompun anesthesia (Ketanest 100 mg/kg body weight, Park Davies, Karlsruhe; Rompun 2%, 1 ml/kg body weight; Bayer, Leverkusen, Germany). For each time point and condition, 2–3 animals were used. Beginning on day 1 after wounding, animals in the treatment group received intraperitoneal injections of embelin at 8 mg/kg or vehicle (10% DMSO, 100 mM Tris–HCl, pH 7.4) in the control group every 48 h over the treatment period. For histological analysis, animals were sacrificed and wound tissues were harvested on days 4, 7, or 10 after wounding. Wound tissue was excised and the wound area bisected in caudocranial direction and the tissue was either fixed overnight in 4% PFA in phosphate-buffered saline (PBS) or embedded in optimal-cutting-temperature O.C.T. compound (Tissue-Tek; Sakura Finetek, Staufen, Germany). Histological analysis was performed on serial sections (5-μm cryosections) from the central region of the wound.

### Morphometric analysis of wounds

Morphometric analysis was performed on digital images using the Imaging Software Lucia G 4.80 (Laboratory Imaging Ltd., Prague, Czech Republic). Microscopy of punch wounds was conducted using a Microscope Eclipse 800E (Nikon, Düsseldorf, Germany). The extent of epithelization and granulation tissue formation was determined on hematoxylin/eosin (H&E)-stained paraffin tissue sections as previously described (Lucas *et al*, 2010). The length of the epithelial tongue was determined as the distance between the epithelial tip and the margin of the wound as defined by the presence of hair follicles in non-wounded skin. This parameter reflects the formation of neoepithelium. In addition, the width of the gap between the epithelial tips reflects wound closure. Granulation tissue was defined as the cellular and vascular tissue that formed underneath the neoepithelium and between the wound margins and above the subcutaneous fat tissue. For quantitative analysis of CD31 or α-SMA expression, the area of three high-power fields (HPF) that stained positive for CD31 or α-SMA within the granulation tissue was calculated.

### Histology and microscopy

Tumor tissue samples were snap-frozen in O.C.T. for storage and further processing. Fresh cryosections of tumors (20 μm) were prepared and fixed in 3% PFA for 10 min, followed by incubation in blocking solution (10% normal goat serum; Invitrogen, Karlsruhe, Germany) and incubation with appropriate primary and secondary antibodies. Cryosections of cutaneous punch wounds (5 μm) were fixed in acetone, endogenous peroxidase was inactivated (0.03% H_2_O_2_, 0.15 M NaN_3_), and unspecific binding sites were blocked with 3% BSA in PBS. Cells were seeded for microscopy on cover slips and fixed with 3% PFA and permeabilized in PBS/0.1% saponin. After blocking (3% BSA/0.1% saponin in PBS), cells were incubated with the appropriate antibodies. Sections were mounted in Mowiol mounting medium (Calbiochem, Schwalbach, Germany) and examined with a motorized inverted microscope (Olympus IX81 or IX71 equipped with Cell^R Imaging Software; Tokyo, Japan) using a 60×/1.45 numerical aperture Planapo oil objective.

### Immunohistochemistry

ECs were stained with rat monoclonal anti-mouse CD31 antibody (PEACAM-1; clone MEC 13.3, 1:500; BD Pharmingen, Heidelberg, Germany) and detected using an Alexa Fluor 488-conjugated polyclonal goat anti-rat antibody (1:500; Invitrogen). Proliferating ECs in tumor tissue were stained with a rat anti-mouse CD105 (endoglin) PE-conjugated monoclonal antibody (1:500, clone: MJ7/18; eBioscience, Frankfurt am Main, Germany). Perivascular cells were stained with Cy3-conjugated mouse monoclonal anti-α-smooth muscle actin antibody (1:1,000, α-SMA clone 1A4; Sigma-Aldrich, Munich, Germany). Mitochondrial morphology and membrane potential were visualized by MitoTracker (RedCMXRos) staining (Invitrogen). For cytochrome c staining, a mouse monoclonal anti-cytochrome c antibody was used (1:500; clone 7H8.2C12; BD Biosciences) and detected with a secondary Alexa Fluor 488-conjugated polyclonal goat anti-mouse antibody (1:500; Invitrogen). For VE-cadherin staining, a monoclonal rabbit anti-VE-cadherin antibody (clone D87F2; Cell Signaling) was used and detected with a secondary Alexa Fluor 488-conjugated polyclonal goat anti-rabbit antibody (1:500; Invitrogen).

### Vascular perfusion, leakiness, hypoxia studies

For vessel perfusion and leakiness studies, 200 μl of FITC-dextran (15 mg/ml in PBS, MW 2,000,000; Sigma-Aldrich) was injected into the tail vein of tumor-bearing mice 30 min before animals were sacrificed. All tumors were excised and snap-frozen in O.C.T for storage and further processing. Vessel leakiness was analyzed using 100× images of tumors from FITC-dextran-injected mice and the percentage of leaky vessels (identified by extravasation into the surrounding tumor tissue) among perfused vessels (dextran- and CD31-positive) was determined. Non-perfused vessels were not included. At least four tumors per group and 5–9 images were used, depending on tumor size.

### Cell viability and cell death measurements

Cells were harvested after treatment and viability was quantified by trypan blue exclusion using an automated cell counter (Countess; Invitrogen) according to the manufacturer's instructions. Cell death was quantified by FACS analysis (FACS-Canto; BD Biosciences) of cells stained with propidium iodide (PI) (0.1 μg/ml in PBS) for 5 min. Alternatively, cells were stained for FACS analysis by the LIVE/DEAD® Far Red Dead Cell Stain Kit “NH2 Assay” (Invitrogen) according to the manufacturer's instructions. Apoptosis was assessed by terminal deoxynucleotidyl transferase dUTP nick-end labeling (TUNEL) using the APO-DIRECT™ Kit (BD Pharmingen) according to the instructions of the manufacturer. Data points represent mean values from at least three independent experiments.

### Clonogenicity assays

1 × 10^4^ cells per well were seeded in 6-well plates and allowed to settle. Cells were then transiently exposed to embelin at the indicated concentrations for 24 h. After recovering for 10 days in fresh medium without embelin, colony formation was assessed by crystal violet staining. Cells were washed in PBS, stained with crystal violet (0.2% in 2% EtOH), and dissolved in 0.2 M sodium citrate and 100% EtOH (1:1).

### Analysis of quiescence by CD105 quantification

Loss of CD105 (endoglin) expression in quiescent ECs was quantified using an APC-conjugated anti-human CD105 antibody (Clone: SN6; eBioscience) by flow cytometry using a FACS-Canto (BD Biosciences).

### Cell cycle analysis

Cell cycle analysis was performed by assessment of the DNA content using propidium iodide. Briefly, cells were fixed using 3% PFA and treated with 100 μg/μl RNase A (ThermoScientific) in PI containing staining buffer (APO-DIRECT™ Kit; BD Pharmingen) for 4 h at 37°C. DNA content was assessed by flow cytometry (FACS Calibur, BD) and further evaluated with flow jow. Data are presented as means ± s.e.m. from at least three independent experiments.

### Cell proliferation analysis

Cell proliferation analysis was performed by the assessment of incorporated EdU using the Click-iT® EdU Alexa Fluor® 488 Imaging Kit or the Click-iT Edu Alexa Fluor 647 Flow Cytometry Assay Kit (Molecular Probes, LifeTechnologies) according to the manufacturer's instructions. Cells were incubated with EdU for 48 h, followed by staining and analysis by microscopy or by flow cytometry, respectively. Data are presented as means ± s.e.m. from at least three independent experiments.

### Respiratory chain performance

Basal oxygen consumption of intact cells, reflecting the metabolism of endogenous substrates, was measured shortly after harvesting using a Clark electrode (Hansatech Instruments Limited, King's Lynn, UK). Following permeabilization with digitonin (0.01%), the oxidation rates of the respiratory chain substrates pyruvate (10 mM, in the presence of 1 mM malate, MPOX), malate (10 mM, in the presence of 10 mM glutamate, MGOX), succinate (10 mM, SOX), and glycerol-3-phosphate (20 mM, GPOX) were determined. To estimate respiration coupled to ATP synthesis, oligomycin (10 μM) was added to inhibit the ATP synthase (complex V). The resulting decrease in respiration rate allows us to calculate the oxygen consumption associated with ATP generation. The remaining respiration rate is an indirect, however, valuable indicator of the proton leak across the mitochondrial inner membrane. The maximal respiratory capacity was estimated by measuring the respiration rate in the presence of the uncoupler FCCP (10 μM). These data were used to calculate a respiratory control ratio and the reserve capacity. This respiratory control ratio reflects the integrity of the inner membrane and was calculated as the ratio of maximal respiratory capacity to proton leak (Rustin *et al*, 1994), whereas the reserve capacity for mitochondrial bioenergetic function was calculated by subtracting the basal respiration rate from the maximal rate.

### Oxygen consumption measurements

Measurement of intact cellular respiration was performed using the XF24 analyzer (Seahorse Bioscience). Cells were treated with 5 μM embelin for 30 min. Prior to the respiration assay, cells were rinsed and cultured in assay medium supplemented with 5 mM glucose, 10 mM sodium pyruvate (Sigma), 2 mM glutamate, and 3% FCS according to the manufacturer's protocol. Cells were incubated at 37°C in a CO_2_-free incubator for 1 h prior to measurement. Oxygen consumption rate (OCR), extracellular acidification rate (ECAR), and proton production rate were measured under basal conditions and in the presence of oligomycin, a complex V inhibitor (1 μg/ml), the complex III inhibitor antimycin (2.5 μM), and mitochondrial uncoupler FCCP (3 μM; Sigma) to assess maximal oxidative capacity. After respiration assay, media were aspirated, wells were washed once with PBS, 25 μl of M-Per lysis buffer (Thermo Scientific) was added, and lysates were analyzed for total protein content using BCA assay (Pierce, Thermo Scientific). Data were normalized to total protein content, and a number of parameters were assessed (Brand & Nicholls, 2011) subsequent to the subtraction of non-mitochondrial respiration (NMR) from each value. ATP production by OxPhos was calculated by multiplying ATP turnover [(basal OCR − NMR)—(oligomycin-inhibited OCR − NMR)] by the established phosphorus/oxygen (P/O) ratio of 2.3 (Brand, 2005). ATP production by glycolysis is considered to have a 1:1 ratio with lactate production, which is measured by PPR (Birket *et al*, 2011). Respiratory reserve capacity = (maximal OCR − NMR) − (basal OCR − NMR).

### Titration with oligomycin

Cell changes in OCR and ECAR were measured on Seahorse XF24 in response to addition of oligomycin in consecutive injections that produced final concentrations of 0.08, 0.4, 0.2, and 1 μM. OCR and ECAR changes were normalized to untreated baseline.

### ATP content

ATP levels in viable cells were quantified using CellTiter-GloTM Luminescent Cell Viability assay kit (Promega) according to the manufacturer's instructions. Lyophilized enzyme/substrate mixtures (100 μl) were transferred to opaque 96-well microplates containing cell lysates. The microplates were then incubated at room temperature for 10 min to stabilize luminescence signals, which were then measured using a Tecan GENios Microplate Reader.

### Mitochondrial membrane potential

The inner membrane potential was estimated using JC-1 as a fluorescent indicator. Cells were incubated with JC-1 (2 μM) for 15 min, and the ratio of green monomer (514-/529-nm excitation/emission maxima) to red J-aggregate (585-/590-nm excitation/emission maxima) was determined by flow cytometry using a FACSCanto cytometer equipped with BD FACSDiva software (Becton Dickinson, Heidelberg, Germany).

### Assessment of proliferation and hypoxia

For tumor cell proliferation, a rabbit Ki67 polyclonal antibody (1:200; Menarini Diagnostics, Berlin, Germany) was used. For hypoxia studies, 200 μl of pimonidazole hydrochloride (15 mg/ml; ArtimmunAnalytik GmbH, Kelkheim, Germany) was injected into the tail vein of tumor-bearing mice 30 min before animals were sacrificed by cervical dislocation. The formation of pimonidazole adducts in hypoxic tumor areas was detected with a FITC monoclonal anti-pimonidazole antibody (1:50, clone 4.3.11.3; ArtimmunAnalytik GmbH).

### siRNA-mediated knockdown

Proliferating HUVECs were transiently transfected with specific XIAP-siRNAs (1: 5′-GGGUUUCUUUAUACUGGUGTT-3′; 2: 5′-GGAAUAAAUUGUUCCAUGCTT-3′) or non-targeting control (scr) with Lipofectamine® RNAiMAX Transfection Reagent (Invitrogen) according to the manufacturer's instructions. Cell viability measurements were performed after 48-h incubation.

### Western Blotting

Whole-cell extracts of proliferating or quiescent HUVECs were prepared by cell lysis in CHAPS buffer (10 mM HEPES pH 7.4, 150 mM NaCl, 1% CHAPS) complete protease inhibitor cocktail on ice for 20 min. Proteins were separated by SDS–PAGE prior to transfer onto nitrocellulose membranes (Protran; Schleicher & Schuell, Dassel, Germany). The following primary antibodies were used in Western blotting: human caspase-9 and caspase-3 (clone 8G10; BD Biosciences), Sirt1 (clone 3H10.2; Millipore), PKM1 (Proteintech), PKM2 (clone D78A4; Cell Signaling), XIAP (clone 48; BD Biosciences), LC3B (clone D11; #3868; Cell Signaling).

### ROS measurement

For the measurement of mitochondrial ROS production, proliferating HUVECs were incubated with 5 μM MitoSOX™ Red mitochondrial superoxide indicator (M36008; Molecular Probes, Invitrogen) at 37°C for 10 min. Cell were washed three times and analyzed by FACS analysis.

### NF-κB activity

ELISAs were performed after nuclear extraction using the TransAM® Nuclear Extract Kit and the TransAM® NFκB p65 Kit (Active motif) according to the instructions of the manufacturer. Read out was performed on an ELISA reader (Anthos HT2) at a wavelength of 450/620 nm.

### Statistical analysis

Results are given as means ± s.d. and were compared using unpaired *t*-tests assuming unequal distribution. A significance level of < 0.05 was considered to be statistically significant; *P <* *0.05; **P <* *0.01; ***P <* *0.001.

## The paper explained

### Problem

There is a huge difference between normal and tumor ECs in terms of proliferation rate and metabolic activity. Such a universal difference is rare in cancer biology. On this basis, the rapidly dividing ECs lining the blood vessels represent an attractive target for cancer treatment. Any compound capable of inducing cell death rather selectively in such proliferating ECs would therefore be valuable in the development of antiangiogenic and thus potentially antitumorigenic drugs. In this manuscript, we provide a novel concept for disrupting tumor neoangiogenesis by targeting mitochondrial activity in angiogenic ECs.

### Results

The benzoquinone embelin acts as a weak mitochondrial uncoupler, and this prevents energy-intensive processes such as proliferation, migration, and capillary tube formation that are indispensible for neoangiogenesis during tumor growth and wound healing. By exhausting the intrinsically low respiratory reserve of proliferating ECs, embelin impairs the angiogenic capacity of proliferating ECs by depleting their energy reserves without adversely affecting quiescent ECs. This useful property was exploited therapeutically to inhibit tumor growth in syngenic and xenograft mouse tumor models and during wound healing. To validate mitochondrial targeting in this context, we demonstrate that neoangiogenesis is impaired in mtDNA mutator mice with defective mitochondrial respiration.

### Impact

Weak mitochondrial uncoupling agents that target proliferating ECs could be clinically valuable to control pathological neoangiogenesis and complement cytostatic drugs in cancer treatment, while minimizing collateral damage to normal quiescent ECs.
