# Supplementary material for: Embelin inhibits endothelial mitochondrial respiration and impairs neoangiogenesis during tumor growth and wound healing
Source: EMBO Mol Med. 2014 Mar 20;6(5):624–39. doi: 10.1002/emmm.201303016 (PMC4023885; doi:10.1002/emmm.201303016)
Supplement: Supplementary file 1 [file emmm0006-0624-sd1.pdf]

Supporting Information Fig. 1 Coutelle *et al.*

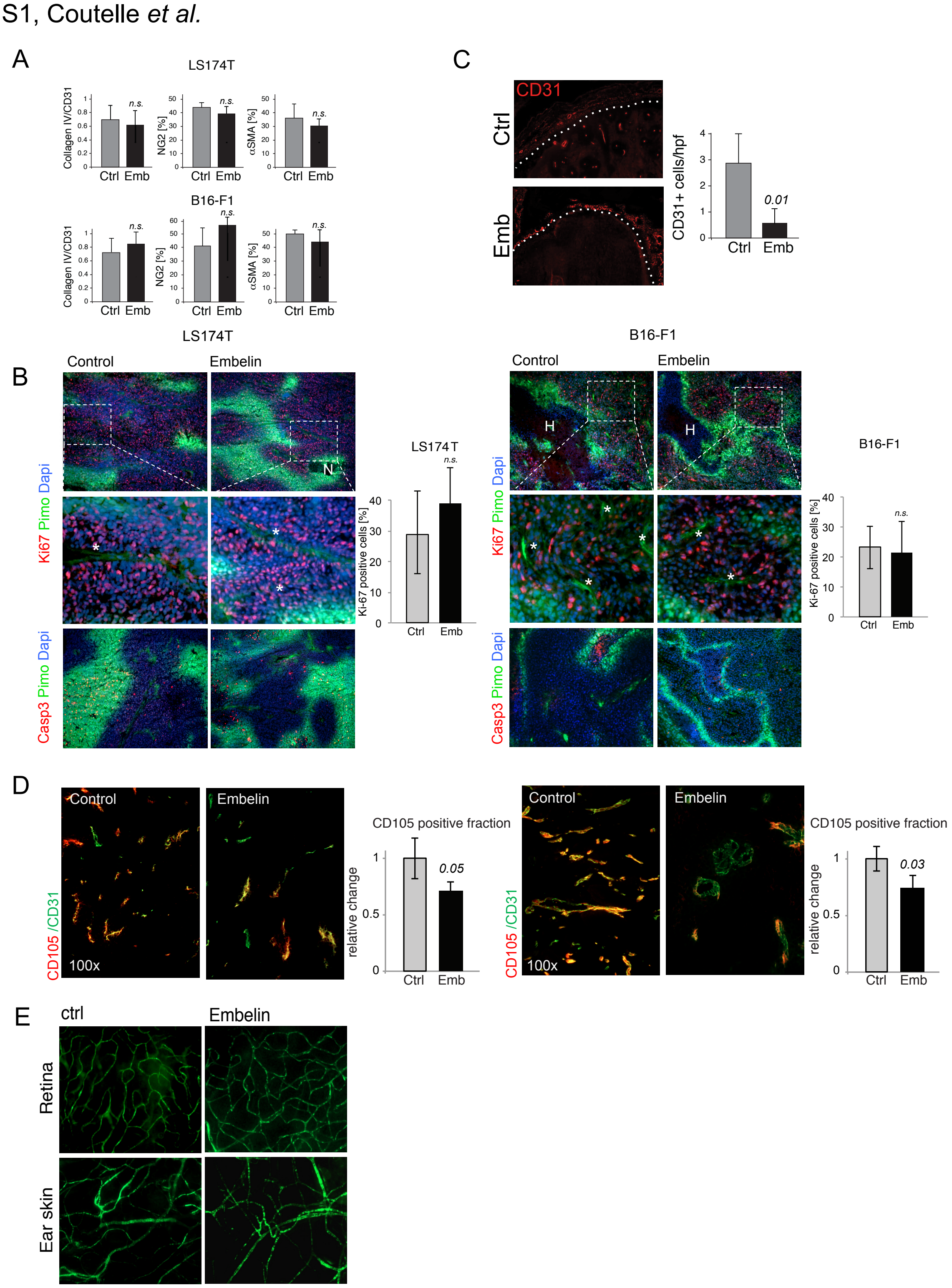

Fig. S1: Embelin lacks direct cytotoxicty *in vivo*.

A. Quantification of collagen IV/CD31 area density and area density of NG2 and SMA pericytes in B16 and LS174T tumors treated as in Fig. 1B.

B. Immunofluorescence analysis of cryosections of LS174T and B16-F1 tumors after a treatment period of 2 weeks with embelin or vehicle (Control) respectively. Areas of hypoxia were detected as pimonidazole adducts and blood vessels (*) were stained with CD31 (green). Ki67 and caspase-3 staining are shown in red. Nuclei were counterstained with DAPI (blue). Quantification of pimonidazole and Ki-67 (lower panel). Values are means ± S.D..

C. Matrigel supplemented with embelin (20 μM) or vehicle (Ctrl) was injected s.c. into recipient mice. Microscopic analysis of CD31 (red). Quantification of CD31 positive blood vessels.

D. Analysis of endoglin expression (CD105 in red) as a marker of angiogenic tumor vessels in LS174T (G) and B16F1 tumors. Quantification of the proportion of angiogenic tumor vessels as the CD105 positive fraction (red) of CD31 positive vessels (green) based on high resolution multiple alignment images (MIAs) of whole tumors (n=5) expressed as mean ± S.D..

E. Microscopic analysis of FITC-Dextran in retina or ear skin from mice treated with embelin or vehicle (ctrl) as described in Fig. 1B.
