# Supplementary material for: Embelin inhibits endothelial mitochondrial respiration and impairs neoangiogenesis during tumor growth and wound healing
Source: EMBO Mol Med. 2014 Mar 20;6(5):624–39. doi: 10.1002/emmm.201303016 (PMC4023885; doi:10.1002/emmm.201303016)
Supplement: Supplementary file 2 [file emmm0006-0624-sd2.pdf]

Supporting Information Fig. 2 Coutelle *et al.*

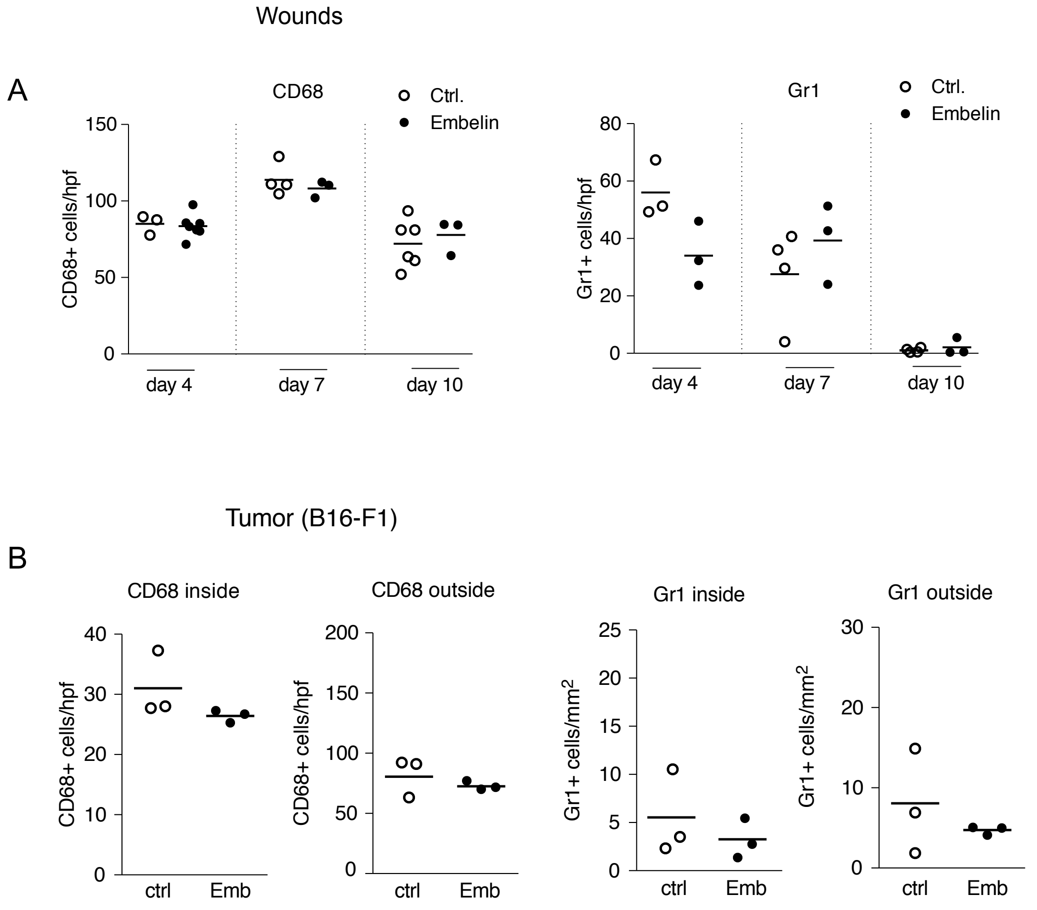

Fig. S2: Quantification of polymorphonuclear neutrophiles (GR1+) and macrophages (CD68+) in punch wounds of B16-F1 tumors of immunocompetent animals

A. Quantification of CD68 and Gr1 staining within granulation tissue in wounds of mice treated as in Fig. 2.

B. Quantification of CD68 and Gr1 staining within granulation tissue in B16-F1 tumors of mice treated as in Fig. 1B.
