# Supplementary material for: Embelin inhibits endothelial mitochondrial respiration and impairs neoangiogenesis during tumor growth and wound healing
Source: EMBO Mol Med. 2014 Mar 20;6(5):624–39. doi: 10.1002/emmm.201303016 (PMC4023885; doi:10.1002/emmm.201303016)
Supplement: Supplementary file 3 [file emmm0006-0624-sd3.pdf]

Supporting Information Fig. 3 Coutelle *et al.*

S3, Coutelle *et al.*

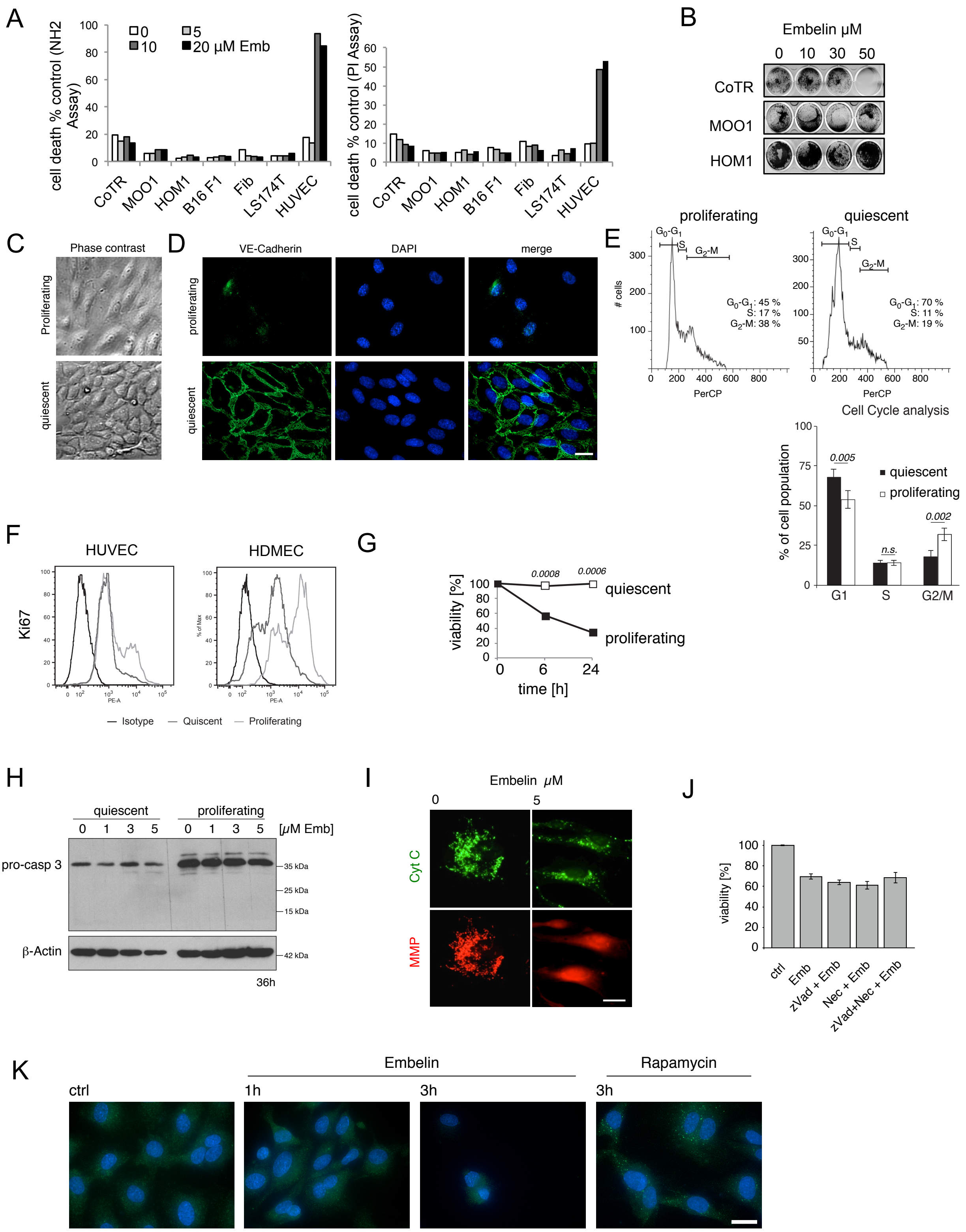

Fig. S3: **Embelin induces cell death specifically in endothelial rather than non-endothelial cell types.**

A. Quantification of embelin-induced cell death in human colonic epithelial cells (CoTr), human primary melanoma cells (MOO1 and HOM1), murine B16-F1 melanoma cells, human dermal fibroblast, human LS174T colorectal cancer cells and proliferating HUVEC. Cell death was measured by the live/dead cell viability assay and propidium iodide assays after 3 hours of exposure to the indicated concentrations of embelin.

B. Clonogenic assays of embelin sensitivity in CoTr, MOO1 and HOM1. Clonogenicity was assessed by crystal violet staining of adherent colonies 14 days after exposure to embelin for 24 hours.

C. Phase contrast microscopy images of quiescent and proliferating cultures of HUVEC.

D. Immunofluorescence staining of quiescent and proliferating HUVEC with anti-VE-cadherin antibody (green). Nuclei were counterstained with DAPI (blue). scale bar = 20 μm

E. Cell cycle analysis of quiescent and proliferating HUVEC measured by FACS analysis of DNA-content. Representative images (upper panel) as well as a summary of three independent experiments (lower panel) are shown. Data points are the mean ± S.E.M.

F. FACS analysis of Ki67 in quiescent and proliferating HUVEC and HDMEC.

G. Measurement of cell viability of proliferating and quiescent HUVEC treated for 24 hours with sublethal dose of embelin (3 μM). Viability was assessed by trypan blue exclusion.

H. Western blot analysis of pro-caspase 3 in whole cell lysates of quiescent and proliferating HUVEC 36 hours after embelin treatment with the indicated concentrations. Actin served as a loading control.

I. Microscopic analysis of proliferating HUVEC showing mitochondrial membrane potential (MMP) by mitotracker (red) and cyt c (green) following embelin treatment (5 μM).
scale bar = 20 μm

J. Measurement of cell viability of proliferating HUVEC treated with embelin (5 μM) after pre-treatment with zVAD (20 μM) or necrostatin (Nec, 30 μM). Viability was assessed by trypan blue exclusion. Data points are the mean ± S.E.M.

K. HUVEC were seeded on cover slides and treated with embelin (5 μM) or rapamycin (40 mg/ml) for the indicated time periods. LC3B was stained with primary rabbit anti LC3B and secondary Alexa Fluor 488 goat anti rabbit antibody. Nuclei were counterstained with Dapi. scale bar = 20 μm
