# Supplementary material for: Embelin inhibits endothelial mitochondrial respiration and impairs neoangiogenesis during tumor growth and wound healing
Source: EMBO Mol Med. 2014 Mar 20;6(5):624–39. doi: 10.1002/emmm.201303016 (PMC4023885; doi:10.1002/emmm.201303016)
Supplement: Supplementary file 4 [file emmm0006-0624-sd4.pdf]

Supporting Information Fig. 4 Coutelle *et al.*

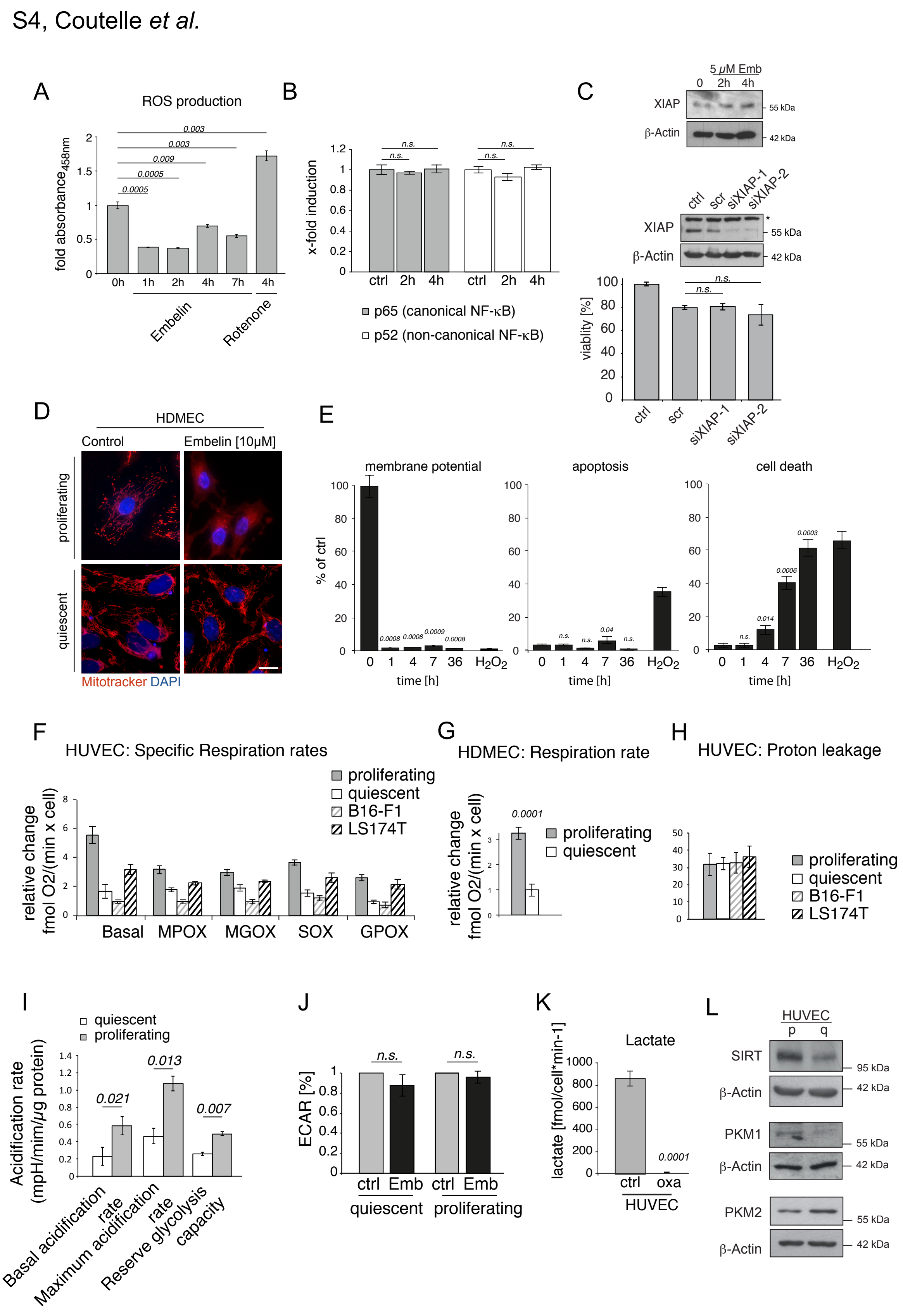

Fig. S4: Sensitivity of EC and tumor cells to to mitochondrial uncoupling agents

A. ROS production was measured in proliferating HUVEC after treatment with embelin (5 μM) by FACS analysis of cells stained with MitoSOX™ Red mitochondrial superoxide indicator. Rotenone (100 nM) served as positive control. Data points are the mean ± S.D..

B. NFϰB activity (p65 and p52) was determined in proliferating HUVEC after treatment with embelin (5 μM) for 2 and 4 hours by ELISA. Data points are the mean ± S.D..

C. Proliferating HUVEC were treated with embelin and whole cell lysates were analysed by Western blotting (upper panel). Proliferating HUVEC were transiently transfected with two specific siRNAs for XIAP or control (scr) and viability was assessed by trypan blue exclusion after 48 hours (lower panel). Knock-down was confirmed by Western blotting. Data points are the mean ± S.D. * marks unspecific bands.

D. Determination of MMP by immunofluorescence analysis of proliferating or quiescent HDMEC using mitrotracker-red staining. Nuclei were counterstained with DAPI.
scale bar = 20 μm

E. Mitochondrial membrane potential (TMRE), apoptosis (Tunel) and cell death (trypan blue exclusion) were measured in proliferating HUVEC treated with embelin (5 μM) or H_2O_2 (2,5 mM, 4h) for the indicated time points. Data points are the mean ± S.E.M..

F. Basal oxygen consumption of HUVEC, reflecting the metabolism of endogenous substrates and the oxidation rates of the respiratory chain substrates pyruvate (10 mM, in the presence of 1 mM malate, MPOX), malate (10 mM, in the presence of 10 mM glutamate, MGOX), succinate (10 mM, SOX) and glycerol-3-phosphate (20 mM, GPOX) were determined.

G. Respiration rate measured in proliferating and quiescent HDMEC.

H. The respiration rate remaining after inhibition of the ATP synthase (Complex V) by oligomycin indicates the proton leak across the mitochondrial inner membrane.

I. Basal rate of extracellular acid efflux predominantly reflecting lactic acid formed during glycolytic energy metabolism (extracellular acid efflux in the presence of 5 mM glucose minus efflux in the presence of glycolysis inhibitor 2 Deoxy-D glucose), maximal acidifcation rate (acid efflux in the presence of oligomycin), and reserve glycolysis capacity (maximal acidification rate – basal acidification rate).

J. Relative extracellular efflux of HUVEC untreated or treated with embelin.

K. Lactate production was measured in HUVEC and HDMEC treated with 20 mM oxamate for 3 hours.

L. Western blot analysis of SIRT, PKM1 and PKM2 in total lysates of quiescent and proliferating HUVEC. Actin served as loading control.
