# Supplementary material for: Embelin inhibits endothelial mitochondrial respiration and impairs neoangiogenesis during tumor growth and wound healing
Source: EMBO Mol Med. 2014 Mar 20;6(5):624–39. doi: 10.1002/emmm.201303016 (PMC4023885; doi:10.1002/emmm.201303016)
Supplement: Supplementary file 5 [file emmm0006-0624-sd5.pdf]

EMBO Molecular Medicine

Embelin inhibits endothelial mitochondrial respiration and impairs neoangiogenesis during tumor growth and wound healing

Oliver Coutelle, Hue-Tran Hornig-Do, Axel Witt, Maria Andree, Lars M. Schiffmann, Michael Piekarek, Kerstin Brinkmann, Jens M. Seeger, Maxim Liwschitz, Satomi Miwa, Michael Hallek, Martin Krönke, Aleksandra Trifunovic, Sabine A. Eming, Rudolf J. Wiesner, Ulrich T. Hacker

Corresponding author: Oliver Coutelle, University of Cologne

Review timeline:

Submission date:	09 May 2013
Editorial Decision:	03 June 2013
Revision received:	02 November 2013
Editorial Decision:	20 November 2013
Revision received:	12 January 2014
Editorial Decision:	22 January 2014
Revision received:	06 February 2014
Accepted:	11 February 2014

Transaction Report:

(Note: With the exception of the correction of typographical or spelling errors that could be a source of ambiguity, letters and reports are not edited. The original formatting of letters and referee reports may not be reflected in this compilation.)

Editor: Roberto Buccione

1st Editorial Decision 03 June 2013

Thank you for the submission of your manuscript to EMBO Molecular Medicine.

You will see that all three Reviewers, while acknowledging the potential interest of your work, raise significant issues that question the conclusiveness of the results and note several technical issues that prevent us from considering publication at this time. I will not dwell into much detail, as the evaluations are detailed and self-explanatory. I would like, however, to highlight a few main points.

Reviewer 1 feels that in general, the conclusions are not sufficiently supported by the experimental data. One main concern in this respect is that the findings rely mostly on the effects of embelin, the specificity of which is far from defined. S/he also finds that there are discrepancies in the outcomes with respect to embelin treatment. Reviewer 1 also notes that the metabolic activities of quiescent vs. proliferating ECs need to be much better investigated. This reviewer lists other important issues that require your attention and action.

Reviewer 2 also feels that the conclusions require further experimentation. Firstly, s/he wonders

why the proliferating cells die, rather than switching metabolism, upon embelin treatment. Reviewer 2 would also like you to verify macrophage and neutrophil recruitment in the tumour models and to include experimentation on mitochondrial uncoupling with alternative approaches. This reviewer also lists other important issues that require your action.

Reviewer 3 is especially concerned that there is a lack of formal demonstration that the experimental approach used does really define, and distinguish between proliferating and non-proliferating cells and feels that the mere removal of growth factors is not sufficient. I agree that this is a critical issue that requires careful thought and action. This reviewer proposes alternative approaches to validate the findings in this respect. Reviewer 3 also points to other important items.

While publication of the paper cannot be considered at this stage, we would be prepared to consider a substantially revised submission, with the understanding that the Reviewers' concerns must be fully addressed with additional experimental data where appropriate and that acceptance of the manuscript will entail a second round of review.

Since the required revision in this case appears to require a significant amount of time, additional work and experimentation and might be technically challenging, I would therefore understand if you chose to rather seek publication elsewhere at this stage. Should you do so, we would welcome a message to this effect.

Please note that it is EMBO Molecular Medicine policy to allow a single round of revision only and that, therefore, acceptance or rejection of the manuscript will depend on the completeness of your responses included in the next, final version of the manuscript.

As you know, EMBO Molecular Medicine has a "scooping protection" policy, whereby similar findings that are published by others during review or revision are not a criterion for rejection. However, I do ask you to get in touch with us after three months if you have not completed your revision, to update us on the status. Please also contact us as soon as possible if similar work is published elsewhere.

***** Reviewer's comments *****

Referee #1 (Remarks):

Summary: In the manuscript ÑMitochondrial targeting inhibits neo-angiogenesis during tumor growth and wound healing" Coutelle and co-workers investigate the effects benzoquinone embelin on angiogenesis in tumors and during wound healing. They report that embelin inhibits tumor growth and wound healing by impairing vascular growth and integrity. They further show that endothelial cells (ECs) are particularly sensitive to embelin leading to increased cell death after treatment. It is claimed that embelin blocks mitochondrial function in proliferating but not quiescent ECs as proliferating ECs use oxidative phosphorylation for energy generation while quiescent ECs do not. The authors conclude that such metabolic targeting could be useful for selective inhibition of pathological angiogenesis.

General judgement: Overall, the manuscript touches an interesting and novel topic with potential medical implications, i.e. the regulation of endothelial metabolism during vascular growth. The authors add to this field by showing that proliferating ECs - contrary to the previous assumptions -

use oxidative metabolism, which makes them more sensitive towards mitochondria-targeting drugs.

While the concept of the manuscript is certainly provocative and novel, the data in the manuscript are in general not sufficient or convincing enough to support the proposed model at this point. For instance, the authors mostly use just one type of pharmacological loss-of-function approach (embelin treatment) whose specificity for mitochondrial function and endothelial metabolism has not been sufficiently characterized. Since embelin is known to target several cellular processes, and the rest of the manuscript is based on this compound, its specificity should have been worked out more carefully. The authors try to address this point by utilizing a mouse model of mitochondrial dysfunction (mutator mouse). However, the significance of the observed phenotypes are also unclear as systemic factors of mitochondrial dysfunction are likely to contribute the impaired vascularization response.

The tumor studies also appear to produce some contradictory results with regards to the effects on tumor cell proliferation and oxygenation: the authors show that embelin treatment has some remarkable inhibitory effects on tumor growth and vascularization, which is however not reflected by the proliferative activity or oxygenation status of the tumor.

An overarching issue is also the writing and packaging of the manuscript. Overall, the manuscript and figures are not well organized, which makes the manuscript difficult to follow - the main text switches back and forth between different figure panels and the introduction is not well connected to the results section.

Specific points - major:
1. The title is too general and needs to be changed - as most of the paper deals with just one type of pharmacological compound (embelin), it is unclear whether the effects of embelin can be generalized to the targeting of mitochondria.

2. The analysis of the metabolic activities in quiescent versus proliferating ECs is incomplete and needs to investigated in much more detail. A systematic analysis of metabolic phenotypes is asked for.

3. How does embelin lead to cell death in ECs? Does it affect the energetic and/or redox state of the cell?

4. How does embelin affect vascular density in vivo? Does it cause lead enhanced EC death? Does it affect EC proliferation or vessel maturation? All these aspects can be analyzed in the tumor sections, e.g. by immunofluorescence staining with the respective marker proteins.

5. How does embelin affect tumor growth rates when it does not affect tumor cell proliferation or apoptosis?

6. The authors justifiably argue that the anti-tumor effect of embelin is due to a reduced vascular supply. However, if this is the case one would expect to see an increase in pimonidazole staining. How can this be explained?

7. What is the effect of embelin on inflammatory cells / macrophages? Non-cell autonomous effects are very likely to contribute to the anti-tumor and anti-angiogenesis effects.

© EMBO

8. It is not entirely clear whether the reduction in mitochondrial membrane potential is the underlying cause of cell death in cultured endothelial cells or whether it is just the consequence of it. A more detailed analysis of the sequence of events would be helpful. In other words: does the reduction in mitochondrial membrane potential precede cell death or does it coincide / follow cell death.

9. The authors state that proliferating endothelial cells operate "near their bioenergetic limit" - how was the respiratory reserve defined / measured?

10. The reduced vascularization response in the aged mutator mice could be the result of many direct and indirect consequences of mitochondrial dysfunction. Without further metabolic and phenotypic characterization of these mice, the results are difficult to interpret. Also, is tumor growth and vascularization altered in these animals?

11. Figure 3B - how was endothelial quiescence assessed in the cell culture assays? A reduction in endoglin (CD105)-positivity is not sufficient to make this point. Since the authors make a strong argument for endothelial quiescence in the response to embelin treatment, quiescence should have been defined characterized more thoroughly.

Specific points - minor:

1. The quality of the imaging in Figure S1B-C is very unsatisfactory, as one cannot observe any details of the vasculature. The authors should provide high-resolution overview and detail images of the respective panels.

2. Results in Figure S1C need be quantified.

3. The statistical summary in figure 5C lacks error bars.

Referee #2 (Remarks):

In this manuscript the authors identified the mechanism by which embelin, a mitochondrial uncoupler, is able to reduce tumor growth and wound healing by inhibiting angiogenesis. Mechanistically, embelin specifically acts on the metabolism of proliferating endothelial cells that, differently from quiescent ECs, utilize mitochondrial oxidative phosphorylation, rather than glycolysis, for energy production. In particular, the increased utilization of the protonic pump results in a reduction of mitochondrial membrane potential and sensitizes to mitochondrial uncouplers, like embelin. Indeed, by exhausting the low respiratory reserve of proliferating ECs, embelin impairs angiogenic capacity of proliferation ECs, without affecting quiescent ECs.

The novelty of these data is due to the discovery that different EC states/phenotypes use different metabolic pathways to achieve energy supply. Based on this, the utilization of mitochondrial uncouplers is promising to specifically target tumor neoangiogenesis, since only 0.01% are proliferating ECs in normal vasculature. However, to further support their model, the authors should address the following points.

Major comments

Overall, it is not clear to the reviewer why proliferating cells should die instead of becoming quiescent in response to embelin. If OxPhos is blocked, won't the cells switch again towards glycolysis and thus become quiescent?

Fig.2B It has been described that embelin has anti-inflammatory activity. Although here a lower dose is used, the authors should still quantify macrophage and neutrophil recruitment, not only in this wound healing model (considering the strong reduction in granulation tissue), but in their tumor models as well, since myeloid cells can strongly influence pathological angiogenesis.

Fig.3B The authors checked cell death upon 3h of embelin treatment at 5 and 10 M. The authors claim the caspase independency of this cell death. However, the apoptotic cascade can be activated at later time points and lower doses. Since in fig. 3C the authors are able to show great differences in tube formation assay also at 1 and 3 M after 36h of treatment, they should check apoptosis in both proliferating and quiescent cells under the same conditions.

There are also some general issues regarding the proposed mechanism of mitochondrial uncoupling. The authors should include some studies with a different mitochondrial uncoupler, like Dinitrophenol (DNP), to show the specificity of the described mechanism.
In line with this, blocking mitochondria at different levels (TCA or respiration) should prevent proliferation and should prevent the effect of embelin. Can the authors prove this?
Furthermore, to ascertain the specific targeting of proliferating ECs, the authors should show that embelin does not have effect on quiescent vessels (for example as shown in figure 6A of Nat Med. 2012 Jul 15. doi: 10.1038/nm.2846).

Fig5 The authors mention an experiment with mitochondrial DNA mutator mice 10 weeks old, which do not bear mitochondrial defects yet. However, only data versus wild type control mice are shown. Please display the data versus the appropriate control 10 week old mutator mice.

Minor comments

Fig S1A: The authors state that there is no pimonidazole staining around FITC-dextran perfused blood vessels. However, the opposite cannot be shown in this staining were both pimonidazole and dextran have the same color. A new staining and quantification needs to clarify this issue. A similar problem arises in Fig.1G and 1H, where one cannot discriminate between an FITC-dextran leakage and a true CD31 positive blood vessel.

Please add when appropriate standard deviation and/or statistics in figures S4B/C/F and 5C.

In the main text, spell out mPTP (mitochondrial permeability transition pore)

On page 4, line 5, the authors mention daily intraperitoneal injections of embelin. Unlikely, according to figure legend and methods, the injections occur every 48h. Please clarify.

Referee #3 (Remarks):

In this MS the authors provide evidences indicating that embelin, a natural weak mithocondrial

uncoupler, inhibits tumor growth. This compound does not target tumor cells but preferentially affect in vivo tumor vasculature. By in vitro experiments the authors suggest that embelin acts on proliferating ECs and affects mitochondrial respiration.

In my opinion the major weakness of the MS is the lack of formal demonstration that experimental approach used really discriminates proliferating and non proliferating cells.

MAJOR POINTS

Fig 2. The authors clearly show that embelin reduces the expression of endoglin. From this result the authors conclude that the drug preferentially targets proliferating ECs. In my opinion this deduction is not fully supported by the experiment and the experiments reported in Fig 3 do not necessarily mirror the in vivo condition. I suggest at least to show proliferative and apoptotic indexes in ECs before and after drug treatment .

In the experiments reported in Figs 3 and 4 the authors study the effect of embelin on proliferating and non-proliferating ECs. I have some doubts that the simple removal of growth factors allows defining proliferating and non proliferating ECs (i.e. How do the authors exclude an autocrine pathway ?). This assumption has to be demonstrated by cell cycle analysis. In my opinion, the best way to arrest EC cycle is the presence of mature cell junctions in confluent ECs (at least 48 hours after reaching confluence). Another option is the block of cell cycle by mitomycin.

By using matrigel based morphogenic assay lasting 36 hours, the authors provide evidences that emblin inhibits the formation of capillary-like structures.
This assay takes into account not only proliferation but almost motogenic events.
Does embelin block EC chemotaxis and chemokinesis induced by angiogenic inducers?
Usually the in vitro double-time of EC in 2D conditions is about 48 hours. Are the authors sure that in matrigel assay ECs are proliferating? How many cells are proliferating?

The results reported in Fig 4 show correlate embelin activity with metabolic activities in proliferating and non proliferating cells. I suggest to measure in the different experimental conditions the levels of ATP, lactate, as well as glucose consumption.

The experiments show in Figure 5 are highly suggestive but they simply demonstrate a crucial role of mytochondria in angiogenesis without any suggestions on the role of respiration.

By results in Fig 1S the authors correctly state that embelin does not modify proliferative and apoptotic indexes of tumor cells. How do the authors explain the reduction burden of tumor growth shown in Fig 1.

1st Revision - authors' response 02 November 2013

Referee #1 (Remarks):

General judgement: Overall, the manuscript touches an interesting and novel topic with potential medical implications, i.e. the regulation of endothelial metabolism during vascular growth. The authors add to this field by showing that proliferating ECs - contrary to the previous assumptions - use oxidative metabolism, which makes them more sensitive towards mitochondria-targeting drugs.

While the concept of the manuscript is certainly provocative and novel, the data in the manuscript are in general not sufficient or convincing enough to support the proposed model at this point. For instance, the authors mostly use just one type of pharmacological loss-of-function approach (embelin treatment) whose specificity for mitochondrial function and endothelial metabolism has not been sufficiently characterized. Since embelin is known to target several cellular processes, and the rest of the manuscript is based on this compound, its specificity should have been worked out more carefully. The authors try to address this point by utilizing a mouse model of mitochondrial dysfunction (mutator mouse). However, the significance of the observed phenotypes are also unclear as systemic factors of mitochondrial dysfunction are likely to contribute the impaired vascularization response.

We thank this reviewer for his/her constructive critique. In the revised manuscript we substantially extend our metabolic analyses of ECs and use additional compounds targeting mitochondrial Oxphos demonstrating the differences between metabolic state of ECs under normal and growth conditions (new Fig.4 and Fig. S4). We analyzed the cytotoxic effects of embelin in more detail (new Fig. S4). We performed additional analyses using mtDNA mutator mice clearly demonstrating that mitochondrial Oxphos is crucial for neoangiogenesis (new Fig. 5).

The tumor studies also appear to produce some contradictory results with regards to the effects on tumor cell proliferation and oxygenation: the authors show that embelin treatment has some remarkable inhibitory effects on tumor growth and vascularization, which is however not reflected by the proliferative activity or oxygenation status of the tumor.
Additional evidence is now provided for more clarity regarding the mode of embelin induced inhibition of tumor growth (new Fig. 1).

An overarching issue is also the writing and packaging of the manuscript. Overall, the manuscript and figures are not well organized, which makes the manuscript difficult to follow - the main text switches back and forth between different figure panels and the introduction is not well connected to the results section.

We apologize for the inappropriate presentation/explanation of our results. The revised manuscript has now been reorganized for a more comprehensible flow of our data.

As requested in his/her **Specific points - major**:

1) *The title is too general and needs to be changed - as most of the paper deals with just one type of pharmacological compound (embelin), it is unclear whether the effects of embelin can be generalized to the targeting of mitochondria.*

In the revised manuscript we provide new evidence that targeting mitochondrial respiration with other weak mitochondrial uncouplers reproduces key aspects of the activity of embelin. Nevertheless to be more specific, we have changed the title of the manuscript:

"Targeting Mitochondrial Respiration Inhibits Neoangiogenesis During Tumor Growth and Wound Healing"

2) The analysis of the metabolic activities in quiescent versus proliferating ECs is incomplete and needs to investigated in much more detail. A systematic analysis of metabolic phenotypes is asked for.

In response to the reviewers' suggestion, we corroborate and extend our metabolic analysis of quiescent *versus* proliferating ECs with new data obtained using a Seahorse XF24 Extracellular Flux Analyzer, which represents a unique opportunity to measure the metabolic status of cells by simultaneously measuring respiration and glycolysis in real-time, and monitor the shift between the two pathways under stress conditions. These analyses confirm and further extend our previous observation using a Clark electrode clearly demonstrating the metabolic shift in ECs under stimulatory/growth condition. In agreement with our previous data an increased respiration rate and lower respiratory reserve capacity is shown in proliferating ECs compared to quiescent ECs (compare Rev#1 Fig. A with Fig. 4F). We now also demonstrate that both cell types retain the principle capacity for glycolysis (new Fig. S4I) although this contributes little to the total ATP pool (new Fig. 4G). Our measurements indicate, that twice as much ATP is produced in proliferating ECs ($p<0.001$) compared to quiescent ECs and that a much greater proportion (>80%) of the ATP pool is produced by oxidative phosphorylation in proliferating ECs ($p<0.001$). In contrast, glycolytic ATP production in both cell types is not significantly different ($p=0.088$) (new Fig. 4G). Accordingly, inhibition of the ATP synthase with oligomycin leads to a greater reduction in the oxygen consumption rate (OCR) in proliferating compared with quiescent cells (Rev#1 Fig. 2) indicating that a greater proportion of the respiratory activity in proliferating EC is linked to ATP generating (coupled) respiration. In the presence of adequate glucose levels both proliferating and quiescent EC retain a limited capacity for compensatory glycolytic activity as illustrated by the increase in ECAR after oligomycin treatment (Rev#1 Fig. 2). However, as mentioned above, this contributes little to the total ATP pool. Overall these findings are consistent with the increased utilization of OxPhos in proliferating ECs.

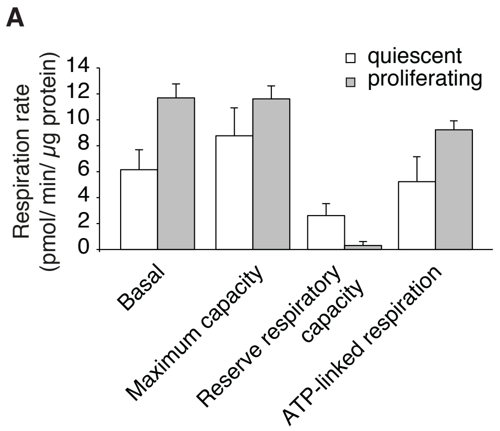

Rev. #1 Fig. A. Respiration rates of quiescent and proliferating HUVEC.

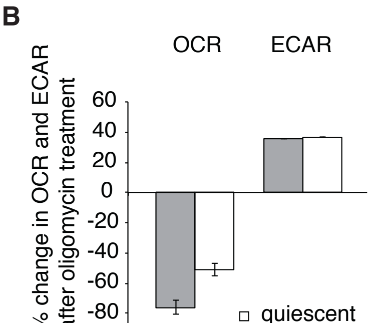

Rev. #1 Fig. B. Fractional change in OCR and ECAR in HUVEC in response to oligomycin. OCR = oxygen consumption rate; ECAR = extracellular acidification rate

3) How does embelin lead to cell death in ECs? Does it affect the energetic and/or redox state of the cell?

We agree that the mode of embelin induced cell death demands further explanation. In the original manuscript we already demonstrated that embelin induced cell death is non-apoptotic (Tunel negative, zVAD independent) (Fig. 3F) and in addition we now show that it is not necroptotic (new Fig. S3J). We also showed that embelin acts as an uncoupler of the MMP (Fig. 4A-B, S4D). In the revised manuscript we provide new evidence linking these observations directly to the energetic state of the cell. We show that more than 80% of the ATP pool is generated by Oxphos in ECs under growth condition (new Fig.4G). Importantly, we now demonstrate that almost all of the ATP *production* by Oxphos is lost following embelin treatment (new Fig. 4G). As a result the ATP *content* in proliferating ECs drops to the level found in quiescent ECs (new Fig. 4I). Despite the principle potential for glycolytic metabolism in proliferating ECs (new Fig. S4I), there is only a very small compensatory increase in glycolytic ATP production (new Fig. 4G) consistent with the lack of increased extracellular acidification (lactate) levels (ECAR) after embelin treatment (new Fig. S4J). We also demonstrate that embelin as well as a further weak mitochondrial uncoupler such as butylated hydroxytoluene (BHT) induce cell death in proliferating but not in quiescent ECs (new Fig. 4E). More importantly, mitochondrial ATP synthase inhibitors, such as oligomycin have a similar effect (new Fig. 4J). These findings suggest that the embelin-induced cell death in ECs under growth condition is the result of ATP depletion.

Addressing the question of embelin induced changes in the redox state of the cell, the production of superoxide by mitochondria was assessed using the MitoSOX™ Red reagent and quantified by FACS analysis. Our measurements showed no increase in ROS production in response to embelin treatment (new Fig. S4A). While we cannot exclude that subtle alterations in ROS levels - as have been implicated in modifying stem cell behavior in mitochondrial mutator mice (Ahlqvist et al., 2012) - could be involved, in ECs we found no evidence for changes in the cellular redox state that could directly account for embelin-induced EC death. In fact, embelin has previously been reported to efficiently scavenge physiologically relevant oxidizing radicals (Joshi et al., 2007). In non-endothelial cell types other modes of action have been reported for embelin including its proposed function as an inhibitor of the X-linked Inhibitor-of-Apoptosis Protein (XIAP) (Nikolovska-Coleska et al., 2004) or as an inhibitor of NFκB activation (Ahn et al., 2007). However, at the comparatively low concentrations required to induce EC cell death, embelin does not alter NFκB signalling (new Fig. S4B) nor does it significantly affect XIAP levels (new Fig. S4C upper panel). In fact, we show that siRNA-mediated XIAP knockdown does not induce cell death in ECs (new Fig. S4C, lower panel). Together these findings suggest that uncoupling mitochondrial respiration is the principle cause of ECs cell death upon embelin exposure under growth conditions.

4) How does embelin affect vascular density in vivo? Does it cause lead enhanced EC death? Does it affect EC proliferation or vessel maturation? All these aspects can be analyzed in the tumor sections, e.g. by immunofluorescence staining with the respective marker proteins.

Our previous data showed that under growth conditions ECs are highly susceptible to embelin treatment whereas quiescent ECs are resistant (Fig. 3E-F). Complementary analyses in our revised manuscript now demonstrate that embelin-induced cell death could be restored in quiescent EC by re-exposing them to growth factors underscoring the specific cytotoxicity of embelin toward proliferating ECs (new Fig. 4D). Together our data suggest that the failure of mitochondria to cover the increased energetic demand of ECs under growth conditions culminates in cell death of ECs when they are induced to proliferate. However, studying the fate of vascular ECs *in vivo* is difficult because the tumor vasculature is embedded and surrounded by tumor cells with vastly higher rates of proliferation and cell death. The lack of specific EC markers of proliferation and cell death makes the distinction between EC and tumor cell proliferation and cell death *in vivo* technically challenging. Indeed evaluation of Ki67 and caspase3 expression in tumor sections showed little EC specific activity even in untreated control tumors, where EC proliferation should be at its maximum (Fig. S1B). However, these findings are consistent with the relatively low proliferation rate of ECs compared to tumor cells and our observation that embelin induced caspase-independent cell death in ECs (Fig. 3F, S3H). CD105 (endoglin) expression has been reported to correlate with the proliferation rate of EC in physiological and pathological neoangiogenesis (Fonsatti et al., 2003; Lebrin et al., 2004). Therefore, the reduced detection of CD105-positive ECs in tumor sections of embelin-treated mice (Fig. S1D) might be the result of increased susceptibility/cell death of proliferating ECs in the tumor microenvironment as conclusively demonstrated in our *in vitro* analyses (Fig. 3).

To assess vessel maturation we now include additional analyses showing that embelin does not significantly alter pericyte recruitment as assessed by the mature pericytes markers αSMA and NG2 in tumors (new Fig. S1A). We already demonstrated that pericyte survival was not adversely affected by embelin treatment *in vitro* (Fig. 3A-B). As capillary sprouts mature into new vessels they become stabilized by Collagen IV positive basement membrane (BM). We therefore examined Collagen IV expression in B16 and LS174T tumors. However, we found no evidence that embelin interfered with the formation of BM (new Fig. S1A). Overall these findings are consistent with embelin induced inhibition of neoangiogenesis.

5) How does embelin affect tumor growth rates when it does not affect tumor cell proliferation or apoptosis?

We thank the reviewer for this important comment and agree that this point was not accurately addressed by the data presented in the original manuscript and apologize for our mistake.
Indeed, the tumor vessel density was significantly reduced following embelin treatment in both tumor models and we agree that this should lead to reduced tumor growth and or increased tumor cell death due to limit oxygen and nutrient supply in embelin treated tumors. We regret that our original evaluation was incorrect and underestimated the extend of hypoxia and cell death for two reasons:

1) Our quantification of tumor hypoxia was mistakenly based on the simple measurement of the area of pimonidazole positive signal in tumor sections. This is inaccurate however, because hypoxic areas are only peripherally stained with pimonidazole (which diffuses from adjacent blood vessels) while the central areas that completely lack perfusion remain pimonidazole negative. This resulted in an underestimation of the hypoxic tumor fraction.
2) On this basis our analysis of cell death and necrosis also needed to be reanalyzed. To avoid regional variation we now evaluated whole tumor sections using high resolution multiple alignment images and manually marked areas of central necrosis for quantification using imageJ software.

The revised quantification of tumor hypoxia and cell death is now presented in the new Fig. 1D. This analysis shows that in embelin treated tumors the reduction in vessel density results in inadequate nutrient and oxygen supply, increased hypoxia and ultimately a greater fraction of tumor cell death or necrosis and these changes could account for the difference observed in the tumor growth rate.

6) The authors justifiably argue that the anti-tumor effect of embelin is due to a reduced vascular supply. However, if this is the case one would expect to see an increase in pimonidazole staining. How can this be explained?

We agree with the reviewer that the reduced vascular density should lead to increased hypoxia and ultimately result in reduced tumor growth and or increased tumor cell death. We regret that our original evaluation was incorrect and underestimated the extent of hypoxia and cell death as we have outlined in response to the previous question.

The revised quantification of tumor hypoxia and cell death is now presented in the new Fig. 1D. This analysis shows that in embelin treated tumors the reduction in vessel density results in increased hypoxia and ultimately a greater fraction of tumor cell death or necrosis and these changes could account for the difference observed in the tumor growth rate.

7) What is the effect of embelin on inflammatory cells / macrophages? Non-cell autonomous effects are very likely to contribute to the anti-tumor and anti-angiogenesis effects.

We agree with the reviewer that non cell-autonomous effects may potentially contribute to anti-tumor and anti-angiogenic effects.
In order to address this point, we now present new detailed histological quantifications of polymorphonuclear neutrophils (PMNs) (GR1+) and macrophages (CD68+) in tumor tissues and punch wounds in immunocompetent C57BL6/J animals. After careful quantification, we found no significant differences in the number of infiltrating macrophages or PMNs observed in the punch wounds of embelin treated animals and controls at four, seven or ten days post injury (new Fig. S2A). In addition, we also conducted similar analyses of PMN and macrophages in the context of syngenic (B16F1) tumors and found that the density of infiltrating macrophages was not significantly altered by embelin treatment, although the intensity of CD68 staining was significantly reduced in comparison to the punch wounds, suggesting that that macrophage activation might be differnt in the context of tumor growth and wound healing (new Fig. S2B). PMN infiltration was noticeably reduced in syngenic tumors, but this was not affected by embelin treatment. We also found no significant differences when comparing macrophage infiltration in the periphery or in the centre of the tumors. On this basis, it seems unlikely that the antiangiogenic effects of embelin treatment in wounds and tumors are secondary to alterations in PMN and macrophage infiltration.

8) It is not entirely clear whether the reduction in mitochondrial membrane potential is the underlying cause of cell death in cultured endothelial cells or whether it is just the consequence of it. A more detailed analysis of the sequence of events would be helpful. In other words: does the reduction in mitochondrial membrane potential precede cell death or does it coincide / follow cell death.

We now present additional data to specifically show that mitochondrial depolarization precedes cell death and is not a consequence of it. Our new time course analysis demonstrates that ECs are already completely depolarized one hour after embelin treatment (5μM), whereas measureable cell death is only observed at this concentration of embelin after 4 hours (new Fig. S4E). Furthermore we now demonstrate that other weak mitochondrial uncouplers such as butylated-hydroxytoluene (BHT) or mitochondrial ATP synthase inhibitors, such as oligomycin potently induce cell death in ECs only under growth conditions (new Fig. 4E, 4J).

9) The authors state that proliferating endothelial cells operate "near their bioenergetic limit" - how was the respiratory reserve defined / measured?

The “respiratory reserve capacity” is used to describe the cellular capacity to further increase oxygen consumption in response to stress or increased workload requiring a sudden increase in energy demand. It represents the difference between the respiration rate at basal and that at maximal respiratory activity i.e. in the presence of the mitochondrial uncoupler (FCCP). The basal rate represents the mitochondrial activity in intact cells, reflecting the metabolism of endogenous substrates prior to addition of compounds used to probe bioenergetic functions. When using the Hansa-Electrode where cells need to be permeabilized the respiratory reserve capacity corresponds to the maximum respiration rate (respiration after addition of succinate and FCCP; SOX max) minus respiration after addition of succinate (Chretien et al., 1994; Rustin et al., 1994).

10) The reduced vascularization response in the aged mutator mice could be the result of many direct and indirect consequences of mitochondrial dysfunction. Without further metabolic and phenotypic characterization of these mice, the results are difficult to interpret. Also, is tumor growth and vascularization altered in these animals?

A detailed characterization of mitochondrial DNA mutator mice including metabolic and phenotypic characterization and tissue functions has previously been reported (Edgar et al., 2009; Ross et al., 2010; Trifunovic et al., 2004). These studies showed that accumulating point mutations in the mitochondrial DNA impair the assembly and stability of the respiratory chain complexes (Edgar et al., 2009). Surprisingly, despite severe respiratory chain dysfunction the premature aging phenotype is not caused by increased ROS production or oxidative stress in mtDNA mutator mice (Trifunovic et al., 2005). Metabolic analyses have shown that high lactate levels in both brain and peripheral tissues are the result of a metabolic shift to a glycolytic or anaerobic condition, where large amounts of lactate are being produced from pyruvate in an environment with increasingly dysfunctional mitochondria (Ross et al., 2010). The reduced potential for neoangiogenesis reported in this study for mtDNA mutator mice and embelin treated animals is consistent with this impairment of OxPhos and the dependence of proliferating ECs on oxidative energy production (new Fig. 4G)
We now provide new evidence that neovascularization is disrupted in aged but not in young mitochondrial mutator mice. To this end, we first demonstrate that matrigel plugs supplemented with VEGF and FGF and implanted for 11 days in young mutator mice (12 weeks old) were as efficiently vascularized as matrigel plugs in wild type animals (including young (12 weeks) and old (30 week) animals) (new Fig. 5A). In addition we now also show that the endothelial networks formed in these plugs have a functional lumen that is capable of conducting FITC labeled dextran into the matrigel plug following tail vein injection of the host animal (new Fig. 5A, middle panel). In contrast, matrigel plugs, implanted into 30 week old mt DNA mutator mice (harbouring mitochondrial dysfunction without obvious phenotypic alteration, see previous citations) were barely vascularized after 11 days, as judged by the amount of FITC dextran staining and histological analysis (new Fig. 5A). To complement these analyses we also show that established vascular networks are not disrupted in old mutator mice (or embelin treated animals) consistent with the specific dependency of neovessel formation on mitochondrial function (new Fig. 5B, S1E).
While we appreciate the reviewers' point regarding tumor growth in aged mutator mice, we regret that due to the very limited number of aged mutator mice available for these experiments and significant variability in tumor growth a statistically sound answer cannot be provided at this point.

11) Figure 3B - how was endothelial quiescence assessed in the cell culture assays? A reduction in endoglin (CD105)-positivity is not sufficient to make this point. Since the authors make a strong argument for endothelial quiescence in the response to embelin treatment, quiescence should have been defined characterized more thoroughly.

As requested, we now provide a more detailed characterization of endothelial quiescence in addition to the CD105 and Ki67 expression shown in the original manuscript (Fig. 3D, S3F). The quiescent phenotype was induced in confluent monolayers of ECs by growth factor withdrawl for 24 hours as previously described (Adams et al., 2005; Kurz et al., 2003; Mahboubi et al., 2001; Mariotti et al., 2006; Vag et al., 2009). Quiescent EC show typical morphological changes in tissue culture, including a cobble-stone appearance that is clearly distinct from the elongated shape of proliferating EC as illustrated in new Fig S3C. Cobblestone cultured HUVECs are in very close contact with each other and most of them stop dividing, become quiescent and go into a resting state comparable to the in vivo epithelial lining of the umbilical veins (Geerts et al., 2011). To confirm this resting state, we examined EdU incorporation by microscopy and flow cytometric quantification to demonstrate the different incorporation rates that distinguish proliferating from quiescent ECs (new Fig.3C). As expected EdU incorporation is significantly lower after growth factor deprivation than in proliferating ECs. Furthermore, contact inhibition of cell growth in quiescent ECs is mediated by vascular endothelial cadherin (VE-cadherin) (Baumeister et al., 2005). We show that VE-cadherin expression is upregulated in cobble-stone cultured ECs confirming their quiescent phenotype (new Fig. S3D).
In addition, we used cell cycle analysis to show the increase in G1 phase and reduction in G2/M phase in growth factor deprived ECs. Cobble-stone cultured ECs demonstrated reduced cell cycle progression in comparison to the corresponding proliferating ECs (new Fig. S3E). The proportion of quiescent ECs in the S phase of the cell cycle distribution was significantly lower than that of the corresponding proliferating cells. Furthermore, significantly more quiescent ECs were distributed in the G0–G1 phases. This indicated that HUVECs subjected to 24h growth factor deprivation were prevented from progressing from G0–G1 to S phase validating our protocol for the establishment of proliferating and quiescent EC cultures.

12) The quality of the imaging in Figure S1B-C is very unsatisfactory, as one cannot observe any details of the vasculature. The authors should provide high-resolution overview and detail images of the respective panels.

We have replaced and extended these figures with high-resolution images accordingly (new Fig. 1D, S1C). To aid orientation, we also provide new overview and detailed images of the respective panels as requested.

13) Results in Figure S1C need be quantified.

A quantification of MVD is now included in new Fig. S1C.

14) The statistical summary in figure 5C lacks error bars.

The quantification of hemoglobin content in the original figure related to the representative matrigel plug depicted in the panel above. In the revised version we have replaced this figure with the new Fig. 5A showing a more appropriate comparison between old *versus* young wild type and old *versus* young mutator mice. The panel includes the whole mounts, histological sections and immunofluorescence images showing FITC dextran perfusion in the matrigel plugs that show more clearly the differences in the vascularization between these experimental groups.

Reviewer #2

The novelty of these data is due to the discovery that different EC states/phenotypes use different metabolic pathways to achieve energy supply. Based on this, the utilization of mitochondrial uncouplers is promising to specifically target tumor neoangiogenesis, since only 0.01% are proliferating ECs in normal vasculature. However, to further support their model, the authors should address the following points.

We thank this reviewer for his/her constructive criticisms. As requested in his/her concerns

15) Overall, it is not clear to the reviewer why proliferating cells should die instead of becoming quiescent in response to embelin. If OxPhos is blocked, won't the cells switch again towards glycolysis and thus become quiescent?

We thank this reviewer for his/her discerning view and regret that the description of these data has been misleading. Our data summarized in our initial manuscript showed that under growth conditions ECs (proliferating ECs) are highly susceptible to mitochondrial uncoupling and die upon exposure to embelin (Fig. 3A-B). We now corroborate and extend our metabolic analyses of quiescent *versus* proliferating ECs with new data obtained using a Seahorse XF24 Extracellular Flux Analyzer which represents a unique opportunity to measure the metabolic status of cells by simultaneously measuring respiration and glycolysis in real-time. These analyses clearly confirm that proliferating ECs increase their respiration rate compared to quiescent ECs (see response to Reviewer#1, question #2). Given that quiescent ECs have a lower energy demand and are relatively resistant to embelin treatment (Fig. 3E-F) growth factor stimulation of quiescent ECs should increase their energy requirement and restore sensitivity to embelin. To test this, we exposed quiescent ECs to embelin after pre-stimulation with VEGF over night and measured cell death as well as cell cycle status. The results clearly show, that the blockade of OxPhos by uncoupling instead of preventing cells from proliferation in respons to growth factor stimulation induced cell death due to the inability to meet the increased energy demands under growth conditions. To further support this, we also show that in embelin treated cells ATP production by OxPhos is nearly abolished (new Fig. 4G and the ATP content therefore drops to the level found in quiescent ECs (new Fig. 4I). Together our data indicate that quiescence is induced in ECs by removing the proliferation signal rather than by restricting its energetic means. However, a metabolic shift in ECs is crucial to cover the cellular demands on energy under growth conditions and thus its alteration results in imbalance in cellular homeostasis and cell death as observed in our analyses.

16) Fig.2B It has been described that embelin has anti-inflammatory activity. Although here a lower dose is used, the authors should still quantify macrophage and neutrophil recruitment, not only in this wound healing model (considering the strong reduction in granulation tissue), but in their tumor models as well, since myeloid cells can strongly influence pathological angiogenesis.

We agree with the reviewer that anti-inflammatory effects of embelin treatment could potentially affect the myeloid compartment and influence pathological angiogenesis. In order to address this point, we now present new detailed histological quantifications of polymorphonuclear neutrophils (PMNs) (GR1+) and macrophages (CD68+) in tumor tissues and punch wounds in immunocompetent C57BL6/J animals.
After careful quantification, we found no significant differences in the number of infiltrating macrophages or PMNs observed in the punch wounds of embelin treated animals and controls at four, seven or ten days post injury (new Fig. S2A). In addition, we also conducted similar analyses of PMN and macrophages in the context of syngenic (B16F1) tumors and found that the density of infiltrating macrophages was not significantly altered by embelin treatment, although the intensity of CD68 staining was significantly reduced in comparison to the punch wounds, suggesting that that macrophage activation might be differnt in the context of tumor growth and wound healing (new Fig. S2B). PMN infiltration was noticeably reduced in syngenic tumors, but this was not affected by embelin treatment. We also found no significant differences when comparing macrophage infiltration in the periphery or in the centre of the tumors. On this basis, it seems unlikely that the antiangiogenic effects of embelin treatment in wounds and tumors are secondary to alterations in PMN and macrophage infiltration.
Notably, while embelin has previously been associated with NF-κB mediated ant-inflammatory activity in human myeloid KBM5 cells (Ahn et al., 2007), in endothelial cells we found no evidence that embelin treatment influenced NF-kB signalling (new Fig. S4B).

17) Fig. 3B: The authors checked cell death upon 3h of embelin treatment at 5 and 10 µM. The authors claim the caspase independency of this cell death. However, the apoptotic cascade can be activated at later time points and lower doses. Since in fig. 3C the authors are able to show great differences in tube formation assay also at 1 and 3 µM after 36h of treatment, they should check apoptosis in both proliferating and quiescent cells under the same conditions.

In response to the reviewers suggestion, we now perform a detailed kinetic and dose response analyses and show that after 36 hours of embelin treatment there is no apoptotic cell death (Tunel staining, new Fig. S4E) and caspase 3 is not cleaved in proliferating or quiescent HUVEC at 1, 3 and 5 µM concentration (new Fig. S3H). However, trypan blue exclusion shows that significant cell death is induced at 5 µM embelin concentration in HUVECs under growth conditions (Fig. 3F). Therefore, as the reviewer suggested, cell death likely contributes to the impaired tube formation response in embelin treated HUVEC (old Fig. 3C), complicating further assessment of embelin mediated effects on proliferation and differentiation in this assay. For this reason we have removed these data from the manuscript.

18) There are also some general issues regarding the proposed mechanism of mitochondrial uncoupling. The authors should include some studies with a different mitochondrial uncoupler, like Dinitrophenol (DNP), to show the specificity of the described mechanism.

In response to the reviewers' suggestion, we now show that in addition to embelin other weak mitochondrial uncouplers such as butylated-hydroxytoluene (BHT) also induce cell death in proliferating but not in quiescent ECs (new Fig. 4E). We also demonstrate that ATP synthase inhibitors, such as oligomycin have a similar effect (new Fig. 4J) indicating that ATP depletion is the underlying cause of cell death in proliferating ECs. In addition we specifically demonstrate that embelin-mediated uncoupling impairs OxPhos-dependent ATP production and as a consequence ATP content drops to the level found in quiescent ECs (new Fig. 4G and I). These findings suggest that proliferating unlike quiescent ECs are critically dependent on oxidative energy production and therefore sensitive to mitochondrial uncoupling.

19) In line with this, blocking mitochondria at different levels (TCA or respiration) should prevent proliferation and should prevent the effect of embelin. Can the authors prove this?

We apologize if this point has not been made sufficiently clear. Our findings show that while OxPhos is required to support the increased energetic demand during proliferation, it is not sufficient for proliferation. Proliferation in ECs is induced by growth factor stimulation and quiescence by removing the proliferation signal - not by a change in the energetic state. Therefore the blockade of OxPhos by uncoupling or blockade of the ATP synthase cannot restore quiescence in angiogenic ECs (as discussed in response to reviewer #1, question #15). The response to mitochondrial blockade differs however, according to the energetic demand. If the energetic demand exceeds the supply then EC death is induced as illustrated by the fact that in quiescent EC resistance to embelin treatment is overcome in response to a proliferative stimulus (Fig. 4D). We also demonstrate that ATP synthase inhibitors (complex V inhibition), such as oligomycin have the same effect (Fig. 4J) indicating that ATP depletion is the underlying cause of cell death in proliferating ECs.

20) Furthermore, to ascertain the specific targeting of proliferating ECs, the authors should show that embelin does not have effect on quiescent vessels (for example as shown in figure 6A of Nat Med. 2012 Jul 15. doi: 10.1038/nm.2846).

We agree that the specificity of embelin for proliferating ECs should result in sparing of mature quiescent blood vessels. To demonstrate this in experimental animals treated with embelin we show that in the easily accessible vascular plexus of the ear and retina we found no obvious morphological differences in adult embelin-treated animals compared with age-matched controls (Fig. S1E). In contrast to the observations made in the tumor vasculature, we found no obvious changes in the microvessel density, vessel diameter and vascular integrity in these tissues in embelin treated animals. Gross morphological inspection of the internal organs also provided no indication for embelin induced vascular disruption of established blood vessels. Although this assessment was not comprehensive, these findings are consistent with embelin's is well tolerated use in traditional medicine (Gupta et al, 1977).

21) Fig 5: The authors mention an experiment with mitochondrial DNA mutator mice 10 weeks old, which do not bear mitochondrial defects yet. However, only data versus wild type control mice are shown. Please display the data versus the appropriate control 10 week old mutator mice.

We thank the reviewer for this suggestion and apologize for our mistake. We agree that a more appropriate comparison should involve young mitochondrial mutator mice rather than wild-type control animals. Accordingly in the new Fig. 5 we now present an expanded panel of matrigel plugs derived from young and old mitochondrial mutator mice as well as young and old wild type controls (Fig. 5A). In addition we now also show that the endothelial networks formed in these plugs have a functional lumen that is capable of conducting FITC labeled dextran into the matrigel plug. Overall these analyses confirm that the defect in neo-vascularization is a function of mitochondrial dysfunction in old mutator mice rather than a consequence of age in general.

22) Fig S1A: The authors state that there is no pimonidazole staining around FITC-dextran perfused blood vessels. However, the opposite cannot be shown in this staining were both pimonidazole and dextran have the same color. A new staining and quantification needs to clarify this issue. A similar problem arises in Fig.1G and 1H, where one cannot discriminate between an FITC-dextran leakage and a true CD31 positive blood vessel.

In old Fig. S1A (now Fig. S1B) both pimonidazole and dextran were deliberately stained in green, to permit additional co-staining for Ki67/Casapase 3 (in red) allowing assessment of its localization in relation to both the tumor vessels and hypoxia. Although we agree with the reviewer that this could in principle cause problems, in this instance this combination was deliberately chosen because the staining patterns are completely different and exclusive. In particular the vicinity of dextran perfused blood vessels is typically well oxygenated and therefore not hypoxic. Although pimonidazole permeates from the vessel lumen, the antibody used for its detection is specific only for the reduced form of pimonidazole that forms in the hypoxic microenvironment and therefore at a distance from the blood vessel lumen not in its oxygen rich vicinity. Therefore there is no overlap in the patterns of dextran and pimonidazole. In addition, the pattern of dextran distribution is clearly different from the pimonidazole pattern – dextran stains the linear or circular lumen of blood vessels or occasionally leaking from the blood vessel lumen, while hypoxia develops as a diffuse pattern that increases specifically with distance from the blood vessel.

We agree with the reviewer, that in the original Fig. 1G and H (now Fig. S1D) the green FITC dextran signal cannot be differentiated from the green CD31 signal, although both could be used to identify and count blood vessels for analysis. Please note, that both FITC and CD31 served to merely identify blood vessels, which where then individually scored for CD105 expression Although this does not hinder detection of the red CD105 signal we have replaced the figures with images from animals that were not FITC dextran injected to avoid confusion.

23) Please add when appropriate standard deviation and/or statistics in figures S4B/C/F and 5C.

We apologize for this oversight. In the revised manuscript we now indicate the statistical significance as requested. Please note that the old Fig. S4B corresponds to new Fig. 4C. The old Fig. S4C has been replaced by a more appropriate comparison of cell death in response to another weak mitochondrial uncoupler (BHT) (new Fig. 4E). The old Fig. S4F corresponds to new Fig. S4G. The original Fig. 5C related to the representative matrigel plug depicted in the panel above. In the revised version we have replaced this Figure with a new Figure 5 showing a more appropriate comparison between old vs young wild type and old vs young mutator mice. The panel includes the whole mounts, fluorescence images and histological sections showing FITC dextran perfusion in the matrigel plugs that show more clearly the differences in the vascularization between these experimental groups (Fig. 5A).

24) In the main text, spell out mPTP (mitochondrial permeability transition pore)

In the reorganization of the revised manuscript this interesting but highly speculative aspect of weak mitochondrial uncouplers is not included anymore due to size limitations.

25) On page 4, line 5, the authors mention daily intraperitoneal injections of embelin. Unlikely, according to figure legend and methods, the injections occur every 48h. Please clarify.

We apologize for this error, which has now been corrected in the revised manuscript stating, that the animals were injected every 48hrs.

Reviewer #3
In my opinion the major weakness of the MS is the lack of formal demonstration that experimental approach used really discriminates proliferating and non proliferating cells.

26) Fig 2: The authors clearly show that embelin reduces the expression of endoglin. From this result the authors conclude that the drug preferentially targets proliferating ECs. In my opinion this deduction is not fully supported by the experiment and the experiments reported in Fig 3 do not necessarily mirror the in vivo condition. I suggest at least to show proliferative and apoptotic indexes in ECs before and after drug treatment.

We thank the reviewer for his comments. In the revised manuscript we have now more thoroughly characterized the quiescent and proliferating EC phenotypes. In particular, we provide additional evidence for quiescence besides endoglin (CD105) and Ki67-expression including phase contrast images (Fig. S3C), assessment of EdU incorporation (Fig. 3C), expression of VE-cadherin adherence junctions (Fig. S3D) and cell cycle analyses (Fig. S3E). To address the reviewers' point concerning the impact of embelin treatment on proliferation and apoptosis, we clearly show that embelin treatment induced significant non-apoptotic (Tunel negative, Caspase negative) cell death in ECs only under growth conditions (Fig. S3H, S4E).
Additional cell cycle analyses of ECs following embelin treatment, were somewhat misleading because they showed a dose-dependent increase in G1 arrested cells with a corresponding reduction in the proportion of cells in G2/M phase (reviewer#3 Fig. A). However, we do not suggest, that embelin treatment leads to growth arrest. In fact the obviously increased proportion of quiescent ECs after embelin treatment is clearly explained by the overwhelming death of proliferating ECs in G2/M. Preferential cell death occurs in 70% of proliferating ECs (30% survived) after 24h of embelin treatment. The calculated increase in G1 arrested cells upon embelin treatment simply reflect the proportion of cells that survive embelin effect (new Fig. S3G). When taking this into account, the data confirm the preferential targeting of proliferating ECs as well as illustrating the resistance of quiescent EC to embelin.

A

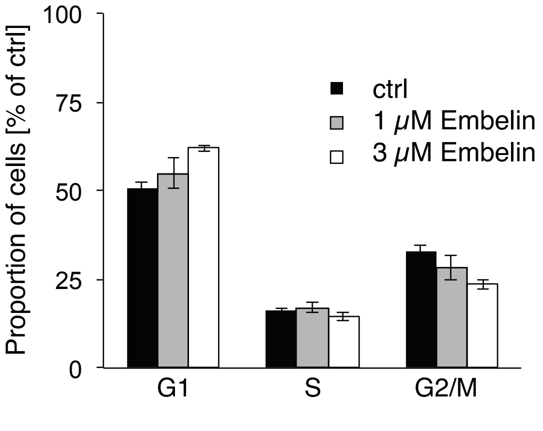

Rev. #3 Fig. A: Cell cycle analysis of proliferating HUVEC stimulated with the indicated concentrations of embelin.

27) In the experiments reported in Figs 3 and 4 the authors study the effect of embelin on proliferating and non-proliferating ECs. I have some doubts that the simple removal of growth factors allows defining proliferating and non proliferating ECs (i.e. How do the authors exclude an autocrine pathway ?). This assumption has to be demonstrated by cell cycle analysis. In my opinion, the best way to arrest EC cycle is the presence of mature cell junctions in confluent ECs (at least 48 hours after reaching confluence). Another option is the block of cell cycle by mitomycin.

The combination of contact inhibition and growth factor withdrawal is typically used to generate quiescent ECs (Adams et al., 2005; Kurz et al., 2003; Mahboubi et al., 2001; Mariotti et al., 2006; Vag et al., 2009). In response to the reviewer, we have verified the quiescent phenotype extensively (see previous question #26) by demonstrating clear differences including the morphological appearance (cobble-stone pattern) (Fig. S3C) in the proliferation rate (EdU incorporation) (Fig. 3C) and the differential expression of VE-cadherin adherence junctions (Fig. S3D). As suggested, we also confirmed by cell cycle analyses that HUVECs subjected to 24h growth factor deprivation were prevented from progressing from G0–G1 to S phase validating our protocol for the establishment of proliferating and quiescent EC cultures (Fig. S3E).

28) By using matrigel based morphogenic assay lasting 36 hours, the authors provide evidences that emblin inhibits the formation of capillary-like structures. This assay takes into account not only proliferation but almost motogenic events. Does embelin block EC chemotaxis and chemokinesis induced by angiogenic inducers?

The reviewer rightly points out, that the *in vitro* assays used to represent the normal extracellular matrix, cannot fully simulate the complex interactions between ECs and other cell types *in vivo*. For these reasons, the *in vitro* angiogenesis assays were intended as a starting point from which to further explore the antiangiogenic activities of embelin. We completely agree with the reviewer, that the tube-forming assay depends not only on proliferation but also motogenic and differentiation events and that a potential effect embelin on chemotaxis and chemokinesis cannot be excluded.
Under growth conditions significant cell death is induced even at low embelin concentration in HUVECs (Fig. 3F and new Fig. S4E). Therefore, cell death likely contributes to the impaired tube formation response in embelin treated HUVEC (old Fig. 3C), complicating further assessment of embelin mediated effects on proliferation and differentiation in this assay. We have therefore removed these data from the manuscript.

29) Usually the in vitro double-time of EC in 2D conditions is about 48 hours. Are the authors sure that in matrigel assay ECs are proliferating? How many cells are proliferating?

We thank the reviewer for this critical comment. As discussed in response to the previous question #28 of this reviewer, our data suggest that under growth conditions significant cell death is induced even at low embelin concentration in HUVECs (Fig. 3F and new Fig. S4E) complicating reliable assessment of embelin mediated effects on proliferation and differentiation in the tube formation assay. We have therefore removed these data from the manuscript.

30) The results reported in Fig 4 correlate embelin activity with metabolic activities in proliferating and non-proliferating cells. I suggest to measure in the different experimental conditions the levels of ATP, lactate, as well as glucose consumption.

We thank the reviewer for this suggestion and now expand our analyses accordingly. In the revised manuscript we now provide new evidence linking embelin-induced EC cell death directly to the energetic state of the cell. We show that more than 80% of the ATP pool is generated by oxidative phosphorylation in proliferating ECs and almost all of the ATP production by OxPhos is lost following embelin treatment (new Fig. 4G). As a result, the ATP content in proliferating ECs drops to the level found in quiescent ECs (new Fig. 4I). Despite the principle potential for glycolytic metabolism production (Fig. S4I), following embelin treatment, there is only a very small compensatory increase in glycolytic ATP production (new Fig. 4G). This observation is in agreement with the lack of increased extracellular acidification/lactate (ECAR) after embelin treatment (Fig. S4J). It seems likely therefore, that cell death in proliferating ECs results from the much greater energy demand compared to quiescent ECs that cannot be met after embelin-induced ATP depletion. In further support of this, we now also show that in addition to embelin other weak mitochondrial uncouplers such as BHT can also induce cell death in proliferating but not in quiescent ECs and importantly, ATP synthase inhibitors, such as oligomycin have a similar effect (Fig. 4E and 4J). These findings suggest that ATP depletion is the principle cause of cell death in embelin treated proliferating ECs.

31) The experiments show in Figure 5 are highly suggestive but they simply demonstrate a crucial role of mitochondria in angiogenesis without any suggestions on the role of respiration.

We agree with the reviewer, that the experimental evidence from the mitochondrial mutator mice demonstrates a crucial role for mitochondria in angiogenesis even if a direct link between respiration and neo-angiogenesis in this model has not yet been demonstrated. Indeed, there is good evidence that the respiratory function in aging mutator mice is severely impaired (Edgar et al., 2009). Although mitochondrial DNA (mtDNA) mutations start to accumulate very early (E13,5), the development of pathological phenotypes in the mtDNA mutator mice does not occur until the accumulated damage reaches a threshold required to develop into pathological change. Whereas previous analyses extensively addressed the impact of mtDNA mutation on the homeostasis and the function of a number of different tissues including heart, liver, muscle, peripheral (cochlea) and central auditory system, no alteration of the endothelial compartment has so far been reported upon accumulation of mtDNA mutation and mitochondrial dysfunction. Our data demonstrate for the first time that accumulation of mtDNA mutation inhibits neo-angiogenesis (new Fig. 5A). In line with these findings we also provide new evidence showing that more than 80% of the ATP production in proliferating ECs is generated by OxPhos (new Fig. 4G), and depletion of the ATP content results in an energy crisis that leads to EC death (Fig 3F). On this basis a specific correlation between mitochondrial respiratory dysfunction and neo-angiogenesis appears plausible and consistent with the inhibitory effects of embelin on neo-angiogenesis during tumor growth and wound healing.

32) By results in Fig 1S the authors correctly state that embelin does not modify proliferative and apoptotic indexes of tumor cells. How do the authors explain the reduction burden of tumor growth shown in Fig 1.

We thank the reviewer for this very important comment and regret that this point was not accurately addressed by the data presented in the original manuscript. The burden of tumor growth is clearly reduced in the presence of embelin (Fig. 1A-B) and since tumor cells are not directly targeted by embelin (Fig. 3A-B, S3A-B) and immune cell infiltrates are not significantly affected by embelin treatment (new Fig. S2) the obvious explanation is the evident antiangiogenic effect of embelin (Fig. 1C). The significant reduction in micro-vessel-density (MVD) in both tumor models is associated with a reduction in oxygen and nutrient supply leading to increased hypoxia and tumor cell death (new Fig. 1D) explaining the reduction in tumor growth.

We apologize that this point was completely missed in the original manuscript because our analysis of hypoxia and cell death was incorrect and underestimated its extend. In particular, the quantification of tumor hypoxia was mistakenly based on the measurement of the area of pimonidazole positive signal in tumor sections. However, because hypoxic areas are only peripherally stained with pimonidazole the central areas that are often necrotic and completely lack perfusion remain pimonidazole negative and these were mistakenly ignored in the original analyses. In our careful re-analyses we also made use of high resolution multiple alignment images (MIAs) to allow analysis of entire tumor sections and avoid regional variation.

The revised quantification of tumor hypoxia and cell death is now presented in the new Fig. S1D along with representative MIAs.

REFERENCES

Adams, R.L., Adams, I.P., Lindow, S.W., Zhong, W., and Atkin, S.L. (2005). Somatostatin receptors 2 and 5 are preferentially expressed in proliferating endothelium. Br J Cancer 92, 1493-1498.

Ahlqvist, K.J., Hamalainen, R.H., Yatsuga, S., Uutela, M., Terzioglu, M., Gotz, A., Forsstrom, S., Salven, P., Angers-Loustau, A., Kopra, O.H., et al. (2012). Somatic progenitor cell vulnerability to mitochondrial DNA mutagenesis underlies progeroid phenotypes in Polg mutator mice. Cell metabolism 15, 100-109.

Ahn, K.S., Sethi, G., and Aggarwal, B.B. (2007). Embelin, an inhibitor of X chromosome-linked inhibitor-of-apoptosis protein, blocks nuclear factor-kappaB (NF-kappaB) signaling pathway leading to suppression of NF-kappaB-regulated antiapoptotic and metastatic gene products. Mol Pharmacol 71, 209-219.

Baumeister, U., Funke, R., Ebnet, K., Vorschmitt, H., Koch, S., and Vestweber, D. (2005). Association of Csk to VE-cadherin and inhibition of cell proliferation. EMBO J 24, 1686-1695.

Chretien, D., Rustin, P., Bourgeron, T., Rotig, A., Saudubray, J.M., and Munnich, A. (1994). Reference charts for respiratory chain activities in human tissues. Clin Chim Acta 228, 53-70.

Desler, C., Hansen, T.L., Frederiksen, J.B., Marcker, M.L., Singh, K.K., and Juel Rasmussen, L. (2012). Is There a Link between Mitochondrial Reserve Respiratory Capacity and Aging? Journal of aging research 2012, 192503.

Edgar, D., Shabalina, I., Camara, Y., Wredenberg, A., Calvaruso, M.A., Nijtmans, L., Nedergaard, J., Cannon, B., Larsson, N.G., and Trifunovic, A. (2009). Random point mutations with major effects on protein-coding genes are the driving force behind premature aging in mtDNA mutator mice. Cell metabolism 10, 131-138.

Fonsatti, E., Altomonte, M., Nicotra, M.R., Natali, P.G., and Maio, M. (2003). Endoglin (CD105): a powerful therapeutic target on tumor-associated angiogenetic blood vessels. Oncogene 22, 6557-6563.

Geerts, W.J., Vocking, K., Schoonen, N., Haarbosch, L., van Donselaar, E.G., Regan-Klapisz, E., and Post, J.A. (2011). Cobblestone HUVECs: a human model system for studying primary ciliogenesis. Journal of structural biology 176, 350-359.

Joshi, R., Kamat, J.P., and Mukherjee, T. (2007). Free radical scavenging reactions and antioxidant activity of embelin: biochemical and pulse radiolytic studies. Chem Biol Interact 167, 125-134.

Kurz, D.J., Hong, Y., Trivier, E., Huang, H.L., Decary, S., Zang, G.H., Luscher, T.F., and Erusalimsky, J.D. (2003). Fibroblast growth factor-2, but not vascular endothelial growth factor, upregulates telomerase activity in human endothelial cells. Arterioscler Thromb Vasc Biol 23, 748-754.

Lebrin, F., Goumans, M.J., Jonker, L., Carvalho, R.L., Valdimarsdottir, G., Thorikay, M., Mummery, C., Arthur, H.M., and ten Dijke, P. (2004). Endoglin promotes

endothelial cell proliferation and TGF-beta/ALK1 signal transduction. EMBO J 23, 4018-4028.

Mahboubi, K., Li, F., Plescia, J., Kirkiles-Smith, N.C., Mesri, M., Du, Y., Carroll, J.M., Elias, J.A., Altieri, D.C., and Pober, J.S. (2001). Interleukin-11 Up-Regulates Survivin Expression in Endothelial Cells through a Signal Transducer and Activator of Transcription-3 Pathway. Lab Invest 81, 327-334.

Mariotti, M., Castiglioni, S., Bernardini, D., and Maier, J.A. (2006). Interleukin 1 alpha is a marker of endothelial cellular senescent. Immunity & ageing : I & A 3, 4.

Ross, J.M., Oberg, J., Brene, S., Coppotelli, G., Terzioglu, M., Pernold, K., Goiny, M., Sitnikov, R., Kehr, J., Trifunovic, A., et al. (2010). High brain lactate is a hallmark of aging and caused by a shift in the lactate dehydrogenase A/B ratio. Proc Natl Acad Sci U S A 107, 20087-20092.

Rustin, P., Chretien, D., Bourgeron, T., Gerard, B., Rotig, A., Saudubray, J.M., and Munnich, A. (1994). Biochemical and molecular investigations in respiratory chain deficiencies. Clin Chim Acta 228, 35-51.

Trifunovic, A., Hansson, A., Wredenberg, A., Rovio, A.T., Dufour, E., Khvorostov, I., Spelbrink, J.N., Wibom, R., Jacobs, H.T., and Larsson, N.G. (2005). Somatic mtDNA mutations cause aging phenotypes without affecting reactive oxygen species production. Proc Natl Acad Sci U S A 102, 17993-17998.

Trifunovic, A., Wredenberg, A., Falkenberg, M., Spelbrink, J.N., Rovio, A.T., Bruder, C.E., Bohlooly, Y.M., Gidlof, S., Oldfors, A., Wibom, R., et al. (2004). Premature ageing in mice expressing defective mitochondrial DNA polymerase. Nature 429, 417-423.

Vag, T., Schramm, T., Kaiser, W.A., and Hilger, I. (2009). Proliferating and quiescent human umbilical vein endothelial cells (HUVECs): a potential in vitro model to evaluate contrast agents for molecular imaging of angiogenesis. Contrast Media Mol Imaging 4, 192-198.

2nd Editorial Decision 20 November 2013

Thank you for the submission of your manuscript to EMBO Molecular Medicine. We have now heard back from the three Reviewers, whom we asked to re-evaluate your manuscript.

You will see that while Reviewer 3 is now supportive, Reviewers 1 and 2 are still not satisfied that the issues raised were adequately addressed.

Reviewer 1 is concerned with the fact that your data contrast with the recent De Bock, Carmeliet paper (Cell 154:651, 2013), which reports that proliferating endothelial cells make ATP through glycolysis and suggest that you preform metabolic flux studies with radioactive tracers to settle the issue. S/he also still not convinced that the model is appropriate and would suggest removing data generated with it from the Ms.

Reviewer 2, notes the insufficient investigation into autophagy as a mechanism for cell death and the lack of analysis of the mechanism of ATP-depletion induced cell death, together with doubts on the specificity of embelin effects.

We have now re-discussed your manuscript in the light of these comments and agree that the Reviewers' points have merits. However, we feel that concerning the issues raised by Reviewer 1, if you discuss the differences with respect to the De Bock, Carmeliet paper (which in part you do) a bit more and clearly recognise and better discuss the limitations of a pharmacological versus genetic approach we would be satisfied. We do agree, however, that the title should be modified according to his/her suggestion. As for Reviewer 2, we also believe that the points are well taken and would thus encourage you to provide the information requested, by carrying out further experimentation if necessary.

Although it is EMBO Molecular Medicine policy to allow a single round of revision only, I am prepared in this case to allow you to submit a re-revised version as outlined above. I believe that ultimately this would strengthen and consolidate your findings.

Acceptance or rejection of the manuscript will depend on the completeness of your responses included in the next, final version of the manuscript.

As you know, EMBO Molecular Medicine has a "scooping protection" policy, whereby similar findings that are published by others during review or revision are not a criterion for rejection. However, I do ask you to get in touch with us after three months if you have not completed your revision, to update us on the status. Please also contact us as soon as possible if similar work is published elsewhere.

I look forward to seeing a revised form of your manuscript as soon as possible.

***** Reviewer's comments *****

Referee #1 (Remarks):

In their revised manuscript, Coutelle and coworkers provide new experiments and data, which address most of my concerns. However, the following issues need further revision / clarification:

1. The authors claim that most of the ATP that is generated by ECs during growth is derived from mitochondrial respiration. These data are in stark contrast to a recent publication by the Carmeliet group (De Bock et al., Cell 2013), which report that most of the ATP in proliferating ECs is produced by glycolysis. Also, inhibition of mitochondrial respiration with oligomycin or antimycin A did not have effects on angiogenic branching in this study. How do the authors explain this discrepancy?

To further validate the relevance of mitochondrial respiration in proliferating endothelial cells, the authors should perform metabolic flux studies with radioactively-labelled tracers (e.g. glucose, glutamine), so that they can follow the "metabolic fate" of glucose / glutamine more accurately.

2. I am still not convinced that the "mutator mouse" is an adequate model to provide evidence for mitochondrial respiration during pathological angiogenesis. As indicated in my previous comments, the mutator mouse phenotypes are very likely confounded by systemic effects of mitochondrial dysfunction and aging - particularly as mitochondrial activity is not specifically inactivated in endothelial cells. These data should therefore be removed from the manuscript.

3. The title of the manuscript is still too general. A better alternative would be: "Embelin inhibits endothelial mitochondrial respiration and impairs neoangiogenesis during tumor growth and wound healing".

Referee #2 (Comments on Novelty/Model System):

Overall the manuscript will have a good impact. Endothelial cell metabolism is a recent topic and little was known so far. The manuscript goes hands in hands with the work by Carmeliet and colleagues. Obviously one could argue that embelin can target all the different cell types when systemically administered - which limits the strength of the conclusions in vivo.

Referee #2 (Remarks):

The authors have addressed all issues raised. Nevertheless, the mechanism of cell death in endothelial cells induced by embelin remains vague. The authors excluded programmed cell death, but they did not investigate the occurrence of autophagy (which might be triggered by energy depletion); neither do they then clearly define the type of cell death as "necrosis". In other words, there is some confusion about the type of ATP-depletion dependent cell death. To the best of my knowledge, energy depletion can trigger caspase activation or autophagy that does not seem to be the case here, at least for caspase activation.

In figure S1D, the authors should include images that are more representative to the displayed data in the according graphs.

Referee #3 (Remarks):

I have no further comments

2nd Revision - authors' response 12 January 2014

Point-by-Point

Referee #1 (Remarks):
In their revised manuscript, Coutelle and coworkers provide new experiments and data, which address most of my concerns. However, the following issues need further revision / clarification:

We thank this reviewer for his/her critical view of our data.

1. *The authors claim that most of the ATP that is generated by ECs during growth is derived from mitochondrial respiration. These data are in stark contrast to a recent publication by the Carmeliet group (De Bock et al., Cell 2013), which report that most of the ATP in proliferating ECs is produced by glycolysis. Also, inhibition of mitochondrial respiration with oligomycin or antimycin A did not have effects on angiogenic branching in this study. How do the authors explain this discrepancy? To further validate the relevance of mitochondrial respiration in proliferating endothelial cells, the authors should perform metabolic flux studies with radioactively-labelled tracers (e.g. glucose, glutamine), so that they can follow the "metabolic fate" of glucose / glutamine more accurately.*

We thank the reviewer for his/her comment regarding the excellent work of De Bock et al., published during the revision process (De Bock et al, 2013). We feel that our results complement the data generated by the Carmeliet group, especially if one considers that sprouting angiogenesis involves distinct endothelial cell populations with different proliferative states and metabolic requirements.
Previous studies have shown that initial sprouting and branching occurs independently of endothelial cell proliferation (Sholley et al, 1984; Ausprunk & Folkman, 1977). In particular, endothelial tip cells lead the way in a branching vessel by probing the environment and migrating toward an angiogenic stimulus with minimal proliferation. A second endothelial subtype, the stalk cell, trails behind the leading tip cell. Their task is to proliferate, elongate the stalk, form a lumen, and connect to the circulation (De Smet et al, 2009). Accordingly, as de Bock showed, sprouting was not inhibited by mitomycin C - an inhibitor of proliferation. Indeed sprouting can progress without cell division, because the driving force for sprout elongation is likely a pulling force exerted by the tip cells, rather than a pushing force originating from proliferating stalk cells (Gerhardt, 2008). Furthermore, a switch from stalk to tip cell differentiation by inhibition of Notch signalling increases vessel branching (Hellström et al, 2007; Suchting et al, 2007; Lobov et al, 2006) confirming that branching is primarily a function of non-proliferative tip cells.
The study by De Bock et al. clearly demonstrates the important role of glycolytic metabolism in non-proliferative tip cells expressing high levels of the glycolytic activator PFKFB3. In agreement with De Bock et al, we show that non-proliferating ECs, which exhibit low respiration rates, have a high rate of glycolysis. Accordingly, exposure of quiescent endothelial cells to oxamate an inhibitor of LDH significantly decreased cell viability confirming the importance of glycolysis in non-proliferating ECs (Fig. 4K). However, as the work by De Bock et al. shows, the metabolic situation is different in proliferating stalk cells, where PFKFB3 expression, and therefore glycolytic energy production is reduced by notch

signalling. In line with this, proliferating EC were not affected by oxamate (Fig. 3F) but sensitive to inhibitors of OXPHOS. Specifically, mitochondrial inhibition with oligomycin or mitochondrial uncoupling with embelin or BHT results in depletion of ATP (Fig. 4I) and thus cell death in proliferating- but not in non-proliferating ECs (Fig. 3F, 4E and J). Indeed, the observation in the De Bock study that inhibition of mitochondrial respiration with oligomycin or antimycin A did not have any effects on angiogenic branching, confirms that branching can occur independently of mitochondrial respiration.

Our extensive biochemical analyses clearly demonstrate that proliferating endothelial cells in addition to glycolysis also employ mitochondrial ATP production during angiogenic growth, while De Bock et al. discount the need for mitochondrial respiration in their Figure 1C based on radioactively-labelled tracers to study glycolytic flux. Despite the widespread use of the [5-^{3}H]glucose detritiation method in the seventies and eighties to determine metabolic flux rates, more recently it has been shown convincingly that rates of glycolysis obtained by quantitation of $^{3}H_2O$ production from [5-^{3}H]glucose may be overestimated (Goodwin et al, 2001). In particular, it has been reported that detritiation of [5-^{3}H]glucose, which occurs in the pentose phosphate pathway, takes place even in the absence of net glycolytic flux, leading to an overestimation of true glycolysis rates. On this basis, we have some doubt as to whether metabolic flux studies with isotopic tracers as suggested by the reviewer could help to resolve these issues. In line with a number of recent publications (Zhong et al, 2010; Birket et al, 2011; Bonnen et al, 2013) we have used microscale oxygraphy analysis to quantify the relative contribution of ATP produced by OXPHOS and glycolysis in each cell type by converting both the oligomycin-sensitive oxygen consumption rate and glycolytic rate into the production rate of ATP (Brand, 2005). This method permits a very reliable estimation of the true ATP production rates attributable to OXPHOS or glycolysis respectively (Fig. 4G).

Based on different models and multiple approaches we have produced consistent data showing that the increased energy demand in proliferating ECs is covered by increased rates of oxidative phosphorylation, and we showed that targeting mitochondrial respiration maybe exploited therapeutically to inhibit tumor angiogenesis.

2. *I am still not convinced that the "mutator mouse" is an adequate model to provide evidence for mitochondrial respiration during pathological angiogenesis. As indicated in my previous comments, the mutator mouse phenotypes are very likely confounded by systemic effects of mitochondrial dysfunction and aging - particularly as mitochondrial activity is not specifically inactivated in endothelial cells. These data should therefore be removed from the manuscript.*

We agree with the reviewer that the mutator model is not without limitations and systemic tissue dysfunction (although not obvious at this age) might in principle contribute to the phenotype observed in these animals. While we cannot formally exclude systemic mitochondrial defects as confounding factors, it seems unlikely that they are major contributors to the defective neovascularization observed for the following reasons. Bone marrow-derived myeloid cells that have an important role in regulating the formation and maintenance of blood vessels in tumors appear to be unaffected in this mouse model given

that hematopoietic stem cell (HSC) frequencies in mutator mice do not differ from those of wild-type mice and evidenced further by an intact production of myeloid cells (Norddahl *et al*, 2011), a good indicator of maintained HSC function (Domen *et al*, 2000). Recent studies have shown that ROS are critically important for VEGF signaling in vitro and angiogenesis in vivo (Ushio-Fukai, 2006). However, despite severe respiratory chain dysfunction the premature aging phenotype is not caused by alterations in ROS production or oxidative stress both potential drivers of angiogenesis (Trifunovic *et al*, 2005). Accordingly, evidence of systemic vascular defects has not been reported in mtDNA mutator mice previously or in this study. On this basis, we feel justified in using matrigel plugs as a valuable technique to examine the capapacity for neoangiogenesis in vivo. Overall, we feel that mutator mouse model further supports the evidence presented for the involvement of mitochondrial OXPHOS in neoangiogenesis and we would like to ask the reviewer to reconsider the value of these findings in light of all the data presented to support this claim. To specifically address the issue raised by the reviewer we are currently developing an endothelial cell specific mouse model of mitochondrial dysfunction.

3. *The title of the manuscript is still too general. A better alternative would be: "Embelin inhibits endothelial mitochondrial respiration and impairs neoangiogenesis during tumor growth and wound healing".*

We have changed the title of our manuscript as suggested.

Referee #2 (Comments on Novelty/Model System):

Overall the manuscript will have a good impact. Endothelial cell metabolism is a recent topic and little was known so far. The manuscript goes hands in hands with the work by Carmeliet and colleagues. Obviously one could argue that embelin can target all the different cell types when systemically administered - which limits the strength of the conclusions in vivo.

We thank this reviewer for his/her discerning view and constructive criticism. We agree, that systemic application of embelin may lead to off target effects confounding the results and this is why we also compared systemic with local application of embelin in matrigel plugs to minimize the impact on non-endothelial cell types (Fig. 1 and S1C). Importantly, we see very similar effects upon local- compared with systemic application of embelin supporting our view that embelin acts primarily and directly on proliferating ECs as a mitochondrial uncoupler that inhibits neoangiogenesis. Furthermore, we found no systemic vascular defects in mice after embelin treatment (Fig S1E). This issue is now discussed in more detail in the discussion.

Referee #2 (Remarks):

The authors have addressed all issues raised. Nevertheless, the mechanism of cell death in endothelial cells induced by embelin remains vague. The authors excluded programmed cell death, but they did not investigate the occurrence of autophagy (which might be triggered by energy depletion); neither do they then clearly define the type of cell death as "necrosis". In other words, there is some confusion about the type of ATP-depletion dependent cell death. To the best of my knowledge, energy depletion can trigger caspase activation or autophagy that does not seem to be the case here, at least for caspase activation.

As requested we performed a detailed analyses of autophagy in embelin-induced cell death. A reliable method for monitoring autophagy and autophagy-related processes, including autophagic cell death is the detection of LC3 by immunoblotting or immunofluorescence (Tanida et al, 2008). Indeed, we found that embelin treatment of ECs leads to the conversion of LC3-I to LC3-II (new Fig. 3G and S3K), however, embelin-induced cell death was only minimally affected when autophagy was inhibited with bafilomycin or pepstatin, or by specific knock-down of ATG5 (new Fig. 3H). Furthermore, embelin induced cell death preceded rapamycin-induced autophagic cell death by almost 20 hours (new Fig. 3H), suggesting that the induction of autophagic cell death is not a major cytotoxic effect of embelin. Nevertheless, given that autophagy is one of the cellular responses to energy depletion due to metabolic stress (Zappavigna et al, 2013) these findings are consistent with our conclusions regarding the effect of embelin exposure on cellular ATP homeostasis.

In figure S1D, the authors should include images that are more representative to the displayed data in the according graphs.

We thank the reviewer for his comment and have updated Fig S1D with more representative images in the revised manuscript.

References

Ausprunk DH & Folkman J (1977) Migration and proliferation of endothelial cells in preformed and newly formed blood vessels during tumor angiogenesis. *Microvasc Res.* **14:** 53–65

Birket MJ, Orr AL, Gerencser A a, Madden DT, Vitelli C, Swistowski A, Brand MD & Zeng X (2011) A reduction in ATP demand and mitochondrial activity with neural differentiation of human embryonic stem cells. *J. Cell Sci.* **124:** 348–58

De Bock K, Georgiadou M, Schoors S, Kuchnio A, Wong BW, Cantelmo AR, Quaegebeur A, Ghesquière B, Cauwenberghs S, Eelen G, Phng L-K, Betz I, Tembuyser B, Brepoels K, Welti J, Geudens I, Segura I, Cruys B, Bifari F, Decimo I, *et al* (2013) Role of PFKFB3-driven glycolysis in vessel sprouting. *Cell* **154:** 651–63

Bonnen PE, Yarham JW, Besse A, Wu P, Faqeih E a, Al-Asmari AM, Saleh M a M, Eyaid W, Hadeel A, He L, Smith F, Yau S, Simcox EM, Miwa S, Donti T, Abu-Amero KK, Wong L-J, Craigen WJ, Graham BH, Scott KL, *et al* (2013) Mutations in FBXL4 cause mitochondrial encephalopathy and a disorder of mitochondrial DNA maintenance. *Am. J. Hum. Genet.* **93:** 471–81

Brand MD (2005) Keilin Memorial Lecture The efficiency and plasticity of mitochondrial energy transduction. *Biochem Soc Trans* **33:** 897–904

Domen J, Cheshier SH & Weissman IL (2000) The role of apoptosis in the regulation of hematopoietic stem cells: Overexpression of Bcl-2 increases both their number and repopulation potential. *J. Exp. Med.* **191:** 253–64

Gerhardt H (2008) VEGF and endothelial guidance in angiogenic sprouting. *Organogesis* **4:** 241–246

Goodwin GW, Cohen DM & Taegtmeyer H (2001) [5-3H]glucose overestimates glycolytic flux in isolated working rat heart: role of the pentose phosphate pathway. *Am. J. Physiol. Endocrinol. Metab.* **280:** E502–8

Hellström M, Phng L-K, Hofmann JJ, Wallgard E, Coultas L, Lindblom P, Alva J, Nilsson A-K, Karlsson L, Gaiano N, Yoon K, Rossant J, Iruela-Arispe ML, Kalén M, Gerhardt H & Betsholtz C (2007) Dll4 signalling through Notch1 regulates formation of tip cells during angiogenesis. *Nature* **445:** 776–80

Lobov IB, Renard RA, Papadopoulos N, Gale NW, Thurston G, Yancopoulos GD & Wiegand SJ (2006) Delta-like ligand 4 (Dll4) is induced by VEGF as a negative regulator of angiogenic sprouting BIOLOGY. **4:** 1–6

Norddahl GL, Pronk CJ, Wahlestedt M, Sten G, Nygren JM, Ugale A, Sigvardsson M & Bryder D (2011) Accumulating mitochondrial DNA mutations drive premature hematopoietic aging phenotypes distinct from physiological stem cell aging. *Cell Stem Cell* **8:** 499–510

Phng L-K & Gerhardt H (2009) Angiogenesis: a team effort coordinated by notch. *Dev. Cell* **16:** 196–208

Sholley M, Ferguson G, Seibel H, Montour J & Wilson J (1984) Mechanisms of neovascularization. Vascular sprouting can occur without proliferation of endothelial cells. *Lab Invest* **51:** 624–634

De Smet F, Segura I, De Bock K, Hohensinner PJ & Carmeliet P (2009) Mechanisms of vessel branching: filopodia on endothelial tip cells lead the way. *Arterioscler. Thromb. Vasc. Biol.* **29:** 639–49

Suchting S, Freitas C, le Noble F, Benedito R, Bréant C, Duarte A & Eichmann A (2007) The Notch ligand Delta-like 4 negatively regulates endothelial tip cell formation and vessel branching. *Proc. Natl. Acad. Sci. U. S. A.* **104:** 3225–30

Trifunovic A, Hansson A, Wredenberg A, Rovio AT, Dufour E, Khvorostov I, Spelbrink JN, Wibom R, Jacobs HT & Larsson NG (2005) Somatic mtDNA mutations cause aging phenotypes without affecting reactive oxygen species production. *Proc Natl Acad Sci U. S. A.* **102:** 17993–17998

Ushio-Fukai M (2006) Redox signaling in angiogenesis: role of NADPH oxidase. *Cardiovasc. Res.* **71:** 226–35

Zappavigna S, Luce A, Vitale G, Merola N, Facchini S & Caraglia M (2013) Autophagic cell death : A new frontier in cancer research. *Advances in Bioscience and Biotechnology* **4**: 250–262

Zhong L, D'Urso A, Toiber D, Sebastian C, Henry RE, Vadysirisack DD, Guimaraes A, Marinelli B, Wikstrom JD, Nir T, Clish CB, Vaitheesvaran B, Iliopoulos O, Kurland I, Dor Y, Weissleder R, Shirihai OS, Ellisen LW, Espinosa JM & Mostoslavsky R (2010) The histone deacetylase Sirt6 regulates glucose homeostasis via Hif1alpha. *Cell* **140:** 280–93

3rd Editorial Decision 22 January 2014

Thank you for the submission of your revised manuscript to EMBO Molecular Medicine. We have now received the enclosed report from the Reviewer that was asked to re-assess it. As you will see s/he is now supportive and I am pleased to inform you that I am ready to formally accept your manuscript pending some minor technical amendments.

***** Reviewer's comments *****

Referee #2 (Remarks):

The authors have addressed and discussed all my concerns
