# Supplementary figures and images for: Embelin inhibits endothelial mitochondrial respiration and impairs neoangiogenesis during tumor growth and wound healing
Source: EMBO Mol Med. 2014 Mar 20;6(5):624–39. doi: 10.1002/emmm.201303016 (PMC4023885; doi:10.1002/emmm.201303016)

Source data Fig. 3G Coutelle et al.

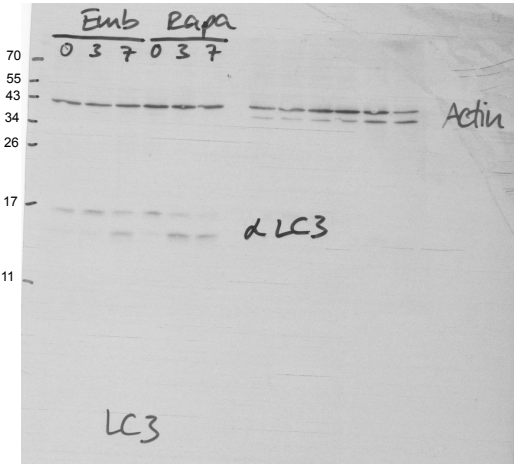

Supplement: Supplementary file 6 [file emmm0006-0624-sd6.pdf]
